# Mark3 a Prognostic Marker for the Endometrial Cancer

**DOI:** 10.3390/curroncol32030157

**Published:** 2025-03-10

**Authors:** Yudan Wang, Liyuan Guo

**Affiliations:** Department of Gynecology Oncology, Harbin Medical University Cancer Hospital, Harbin 150081, China; 202101450@hrbmu.edu.cn

**Keywords:** endometrial cancer, gene enrichment analysis, MARK3, Transwell assay, Western blot

## Abstract

**Introduction:** Endometrial cancer (EC) is one of the most common gynecologic cancers, with an increasing incidence due to variables such as aging and lifestyle changes. Current biomarkers exhibit limited prognostic value, despite advancements in understanding their molecular basis, underscoring the necessity for new molecular markers. Microtubule affinity-regulating kinase 3 (MARK3) has been identified as a potential candidate owing to its established prognostic significance in various cancers; however, its function in endometrial cancer (EC) is not yet well understood. **Methods:** This study investigates the function of MARK3 in endometrial cancer through the analysis of Ishikawa and HEC-1B cell lines. A series of assays were conducted, including colony formation, CCK-8 viability, EDU proliferation assays, scratch wound healing tests, and Transwell migration assays, to investigate the effects of MARK3 overexpression. We conducted RT-qPCR, Western blot, and immunofluorescence assays to evaluate the molecular mechanisms influencing cell proliferation and migration. Bioinformatics analysis utilized publicly available datasets to examine the gene enrichment and co-expression networks. **Results:** The overexpression of MARK3 markedly reduced colony formation in both Ishikawa (*p* = 0.0039) and HEC-1B (*p* = 0.0014) cell lines. Furthermore, the overexpression of MARK3 led to decreased cell viability, as demonstrated by the EDU assay results (Ishikawa-OE *p* = 0.0302; HEC-OE *p* = 0.0037). The molecular analysis supported these findings, indicating an increase in phosphorylated AKT (pAKT), thereby suggesting MARK3’s role in regulating cell survival pathways. Gene enrichment analysis revealed pathways associated with cell cycle regulation and apoptosis, whereas co-expression analysis pinpointed critical interacting genes that may play a role in EC progression. **Conclusions:** MARK3 is essential in the regulation of cell proliferation and migration in endometrial cancer, positioning it as a potential prognostic biomarker and therapeutic target. This study represents the inaugural investigation into the functional role of MARK3 in endothelial cell progression, thereby enhancing our comprehension of its mechanistic influence on cancer biology and its implications for personalized therapy. Bioinformatics analysis reinforces the relevance of MARK3 in endometrial cancer, offering new insights into its clinical significance.

## 1. Introduction

Endometrial cancer (EC) is a prevalent gynecological malignancy in developed countries, accounting for over 7% of all cancers in women and approximately 3% of female cancer-related fatalities globally. EC is the most common gynecological malignancy in high-income countries and is increasing in incidence [1]. EC predominantly affects postmenopausal women, with over 90% of cases occurring in individuals aged 50 and above. The lifetime risk of developing EC is estimated to be 2.81%. Early detection of the disease in postmenopausal women is often achievable in many cases with characteristic abnormal uterine bleeding [2]. However, 20–30% of patients may progress to advanced or recurrent disease, with deteriorating prognosis. The five-year survival rate of EC patients with localized tumor is 95%, decreasing to 69% for those with regional lymph node involvement and further decreasing to 17% for patients with distant metastasis. This underscores the necessity for discovering reliable prognostic biomarkers to facilitate clinical management [3,4].

Characteristic clinical presentations of EC include abnormal vaginal bleeding, abnormal vaginal discharge, and pelvic pain. Diagnosis in premenopausal women is often more difficult, as abnormal bleeding can be misinterpreted as menstrual irregularities [5]. The standard diagnostic methods for EC include endometrial biopsy for pathology confirmation and transvaginal ultrasonography (TVUS) for assessing endometrial thickness. When biopsy results are inconclusive, hysteroscopy may be employed for additional evaluation. Molecular testing, which encompasses analyses of microsatellite instability and mismatch repair proteins, has been increasingly utilized to estimate the prognosis [6,7].

Endometrial cancer is categorized into two primary types according to clinical and molecular features. Type I (endometrioid) endometrial carcinoma, which constitutes 80–90% of cases, is estrogen dependent and often associated with a favorable prognosis [4,8]. These tumors often harbor mutations in PTEN, PIK3CA, and KRAS genes. Type II (non-endometrioid) cancers, including serous and clear cell subtypes, are often with increased aggressiveness with higher incidence of TP53 mutations, chromosomal instability, and high-grade histology. These factors are generally associated with a poorer prognosis and varied therapeutic responses [9].

Microtubule affinity-regulating kinase 3 (MARK3), has become an important target in cancer research. MARK3 is essential for regulating many cellular biology processes, such as polarity, apoptosis, and cell cycle progression, via the phosphorylation of cytoskeletal proteins. Dysregulation of MARK3 has been identified in multiple cancers and has been proven to be associated with tumor progression, metastasis, and treatment resistant [10].

This study investigated the role of MARK3 in endometrial cancer. Gene enrichment analysis identified the molecular pathways associated with MARK3, and co-expression analysis revealed the genes and molecular networks interacting with MARK3 in the context of EC. Endometrial cancer cell lines, Ishikawa and HEC-1B, were utilized to evaluate the functional effects of MARK3 overexpression on critical cellular processes including proliferation and migration. Multiple assays, such as colony formation, CCK-8 viability assays, EDU proliferation assays, scratch wound healing tests, and Transwell migration assays, were conducted to assess the role of MARK3 on EC cells. We also performed RT-qPCR, Western blot, and immunofluorescence assays to examine the changes in molecular pathways that are associated with MARK3.

By exploring the role of MARK3 in endometrial cancer cell, we aimed to gain a deeper understanding of the pathogenesis of EC and shed light upon discovering novel therapeutic options.

## 2. Materials and Methods

### 2.1. Data Acquisition

Gene expression data for endometrial cancer specimens were obtained from The Cancer Genome Atlas (TCGA). The collection comprises RNA sequencing data and clinical information for 547 patients with endometrial cancer. The expression levels of MARK3 were obtained for further analyses.

### 2.2. Correlation with Co-Expressed Genes

Correlation analysis was conducted to explore the relationship between MARK3 and other co-expressed genes. The cor.test function in R calculates Pearson correlation coefficients. Genes exhibiting a correlation coefficient exceeding 0.6 and a *p*-value below 0.05 were deemed significantly correlated with MARK3.

### 2.3. Functional Enrichment Analysis

#### 2.3.1. Gene Ontology Enrichment

We performed Gene Ontology enrichment analysis on the co-expressed genes with MARK3 utilizing the clusterProfiler R package (3.20). The analysis was conducted using the criteria of *p*-value < 0.05 and q-value < 0.05. This step sought to uncover highly enriched biological processes, molecular activities, and cellular components linked to the co-expressed genes.

Bar plots were created, with the y-axis denoting the enriched GO terms, the x-axis reflecting the quantity of genes enriched in each GO term, and the color of the bars signifying the statistical significance of the enrichment. Bubble plots were generated to illustrate the enrichment, with the y-axis denoting the enriched GO terms, the x-axis reflecting the gene ratio (the proportion of genes enriched in each GO term), the circle size representing the gene count, and the circle color indicating the degree of statistical significance.

A circular diagram was created to illustrate the hierarchical links among the GO types. The outermost circle depicted the GO word IDs, categorized into three primary classifications: Molecular Function (MF), Biological Process (BP), and Cellular Component (CC). The second circle illustrated the quantity of genes enriched in each GO word, with colors denoting statistical significance. The third circle illustrated the quantity of overlapping genes associated with each GO term, whereas the innermost circle denoted the fraction of genes inside that category.

#### 2.3.2. KEGG Pathway Enrichment Analysis

KEGG pathway enrichment analysis was conducted on the co-expressed genes utilizing the clusterProfiler R package, with filters applied for pathways exhibiting *p*-values < 0.05 and q-values < 0.05. The objective was to identify key biological pathways potentially implicated in the tumorigenesis process.

Bar plots were created, with the y-axis indicating the enriched pathways and the x-axis displaying the number of genes enriched in each pathway. The color of the bars indicated the statistical significance of the pathway enrichment.

Bubble plots were generated, with the y-axis indicating the enriched KEGG pathways and the x-axis representing the gene ratio for each pathway. The bubble size corresponded to the quantity of genes enriched in each pathway, whereas the bubble color denoted the level of statistical significance.

### 2.4. Cell Lines Cultures

The Ishikawa and HEC-1B endometrial cancer cell lines were purchased from the Procell Life Science & Technology Co., Ltd. in Wuhan, China. Cells were cultured in appropriate media containing 10% fetal bovine serum (Pricilla CM-0283 and CM-0100) and incubated at 37 °C in a humidified environment with 5% CO_2_.

### 2.5. Cell Culture and Transfection Assay

Cryopreserved cells went through rapid thawing in a 37 °C water bath, followed by centrifugation at 800 rpm and resuspension in 1 mL of culture medium. Cells were placed in culture flasks and incubated at 37 °C with 5% CO_2_. Transfection was performed utilizing the jetPRIME reagent (Polyplus 101000046, Polyplus, Illkirch-Graffenstaden, France) in accordance with the manufacturer’s protocol. Lentiviral packaging was completed in 293T cells and infected the target cells in the presence of Polybrene. Successful transfection was confirmed by puromycin (Biosharp BL528A, Biosharp, Hefei, China) selection after 48–72 h.

### 2.6. Colony Formation Assay

The Ishikawa and HEC-1B endometrial cancer cell lines were cultured in six-well plates at a low density of 500 cells per well to provide adequate space for colony development. Cells were cultured in complete media and incubated at 37 °C in a humidified atmosphere containing 5% CO_2_. In the groups, cells transfected to overexpress MARK3 (Ishikawa-OE and HEC-OE) were included, alongside non-overexpressing control cells (Ishikawa and HEC). After a 14-day incubation period, during which colonies reached a visible size, cells were washed with phosphate-buffered saline (PBS) (Solarbio P1020, Solarbio, Beijing, China) and subsequently fixed with 4% paraformaldehyde (Biosharp BL539A) for 15 min. Following fixation, colonies were subjected to staining with 0.5% crystal violet (Solarbio C8470) for 20 min at room temperature. The plates were rinsed with distilled water to eliminate excess stain and subsequently air-dried. Colonies comprising 50 or more cells were enumerated using a microscope to assess colony formation. The assay was conducted in triplicate, and the results were analyzed to evaluate the effect of MARK3 overexpression on colony-forming efficiency in both cell lines.

### 2.7. RT-qPCR Analysis

RNA was extracted from cells utilizing the TIANGEN RNA extraction kit (DP451, Tiangen Biochemical, Beijing, China), and cDNA synthesis was subsequently performed using the TIANGEN reverse transcription kit (KR118-02). Real-time PCR was performed utilizing SYBR fluorescent dye (YESEN 11201ES08, Shanghai, China), with GAPDH serving as the internal control for normalization.

### 2.8. CCK-8 Assay for Cell Viability

CCK-8 was utilized to evaluate cell viability. Cells were plated in 96-well plates and treated with CCK-8 reagent (TargetMOI C0005) for incubation. Cell viability was quantified by measuring absorbance at 450 nm with a microplate reader.

### 2.9. Scratch Wound Healing Assay for Migration

A scratch wound healing assay was conducted to assess cell migration. A sterile pipette tip was employed to induce a scratch in confluent monolayers of cells cultured in 6-well plates. Cells underwent washing to eliminate debris and were subsequently incubated in serum-free media. Images were obtained at 0, 24, 48, and 72 h following the scratch, and migration rates were determined by assessing the wound area at each time interval.

### 2.10. Transwell Assay Migration

A Transwell assay was performed utilizing chambers featuring an 8 μm pore polycarbonate membrane (Corning 3422, Corning, NY, USA). Cells were placed in serum-free media in the upper chamber, while the lower chamber contained media with 10% FBS as a chemoattractant. Following a 24 h incubation period, non-migrated cells were eliminated, while cells that migrated or invaded through the membrane were fixed, stained with crystal violet, and subsequently counted using a microscope.

### 2.11. EDU Assay for Cell Proliferation

An EDU (5-ethynyl-2′-deoxyuridine) incorporation assay was conducted to assess cell proliferation. Cells were given a treatment of 10 µM EDU (Beyotime C0071S, Shanghai, China) for a duration of 2 h, followed by fixation and staining with a Click EDU Imaging Kit. Cells labeled with fluorescent markers were examined using a fluorescence microscope, and the proliferation rate was determined as the percentage of EDU-positive cells.

### 2.12. Western Blot Analysis

Protein samples were obtained through the lysis of cells using RIPA buffer (Biosharp BL504A). Lysates underwent centrifugation, and protein concentrations were determined via the BCA (Beyotime P0010S) assay. Proteins were separated via electrophoresis (Solarbio T1070), followed by transfer to PVDF (Merck ISEQ00010, Darmstadt, Germany) membranes. Membranes underwent overnight incubation with primary antibodies targeting MARK3 (abcam ab52626, Boston, MA, USA), AKT (Zenbio 342529, Chengdu, China), p-AKT (Zenbio 310021), P-PI3K (Abmart TA3242, Shanghai, China), PI3K (Abmart TA6241), p-mTOR (Proteintech 67778-1-IG, Rosemont, IL, USA), mTOR (Proteintech 66888-1-IG), GAPDH (Affinity AF7021-50 µL, Changzhou, China), and ACTIN (Affinity AF7018), subsequently followed by incubation with secondary antibodies (Affinity S0001-100 µL and S0002-100 µL). Detection utilized ECL chemiluminescence (Meilunbio MA0186-1, Dalian, China), with grayscale values quantified to assess protein expression levels.

### 2.13. Immunofluorescence Assay

The immunofluorescence assay (IF) initiates with the seeding of 1 × 10^5^ cells per well in a 24-well plate, followed by an overnight incubation period for cell growth. The supernatant was subsequently removed, and the cells were washed with PBS for three minutes, repeated three times. Subsequently, 300 µL of 4% paraformaldehyde is added to each well for fixation, followed by a 40 min incubation period. The fixative was removed, and the cells were rinsed with PBS for 5 min, repeated three times. Permeabilization involves the addition of 1 mL of permeabilization (Beyotime P0097) solution to each well, followed by incubation at room temperature for 20 min. This is succeeded by washing with PBS for 5 min, repeated three times.

The cells were subsequently incubated with a blocking solution (Boster AR0009, Wuhan, China) for a duration of 30 min. Following the removal of the blocking solution, the primary antibody, diluted in a suitable buffer (typically 1:100), is applied, with a minimum volume of 300 µL per well, and incubated overnight in a humidified chamber. The primary antibody is eliminated, and the cells are washed with PBS for a duration of 10 min, repeated three times. A fluorescently labeled secondary antibody (minimum 300 µL per well) is applied, and the cells are incubated in the dark for one hour. The secondary antibody is eliminated, and the cells are washed with PBS for 10 min, repeated three times. DAPI (Beyotiome P0131) staining is conducted for 10 min, followed by three washes with PBS, each lasting 3 min. The samples were either stored in light-protected conditions or imaged immediately. This protocol guarantees consistent and reliable outcomes for cellular analysis via immunofluorescence.

## 3. Results

### 3.1. MARK3 as a Key Regulator in Endometrial Cancer Proliferation and Associated Pathways

**Figure 1:**
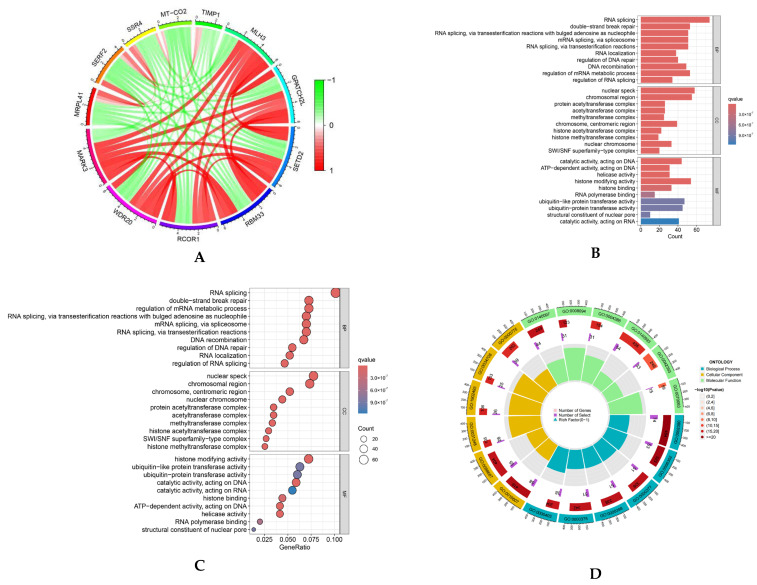

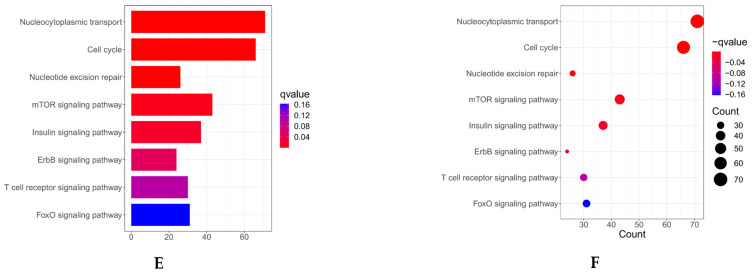
(**A**) Co-expression analysis of MARK3 with other genes in endometrial cancer. Positive correlations are represented by red, while negative correlations are represented by green, indicating the relationships between MARK3 and other genes in the context of endometrial cancer (Supplementary S1). (**B**–**D**) Gene Ontology (GO) enrichment analysis of MARK3 co-expressed genes. The enriched biological processes include cell division and microtubule organization, processes critical for cancer cell proliferation and metastasis. The respective *p*-values and q-values are shown (Supplementary S2). (**E**,**F**) GO and KEGG pathway enrichment analysis of MARK3 co-expressed genes. The analysis identifies key pathways, including cell cycle control and microtubule-based activities, which are essential for carcinogenesis. The associated *p*-values and q-values are presented.

Correlation analysis revealed multiple genes exhibiting substantial positive correlations with MARK3 (correlation coefficient > 0.6, *p*-value < 0.05), suggesting that these genes may possess analogous regulatory mechanisms or biological processes essential to tumor development. A circular map (Figure 1A) was employed to illustrate the coordinated expression patterns of MARK3 and its co-expressed genes in TCGA endometrial cancer samples.

We performed Gene Ontology (GO) enrichment analysis to elucidate the functional roles of these co-expressed genes. This analysis demonstrated that these genes were markedly enriched in biological processes, including cell division and microtubule structure (Figure 1B–D), which are critical for cancer cell proliferation and metastasis. Furthermore, KEGG pathway enrichment analysis identified essential pathways linked to MARK3 co-expressed genes, such as cell cycle control and microtubule-based activities (Figure 1E,F), which are vital for carcinogenesis. The findings indicate that MARK3 may be pivotal in the molecular pathways underlying endometrial cancer growth.

The bioinformatics results highlight the significance of MARK3 in the course of endometrial cancer, positioning it as a viable candidate for additional functional validation and therapeutic targeting.

### 3.2. MARK3 Inhibits the Proliferation of Endometrial Cancer Cells

**Figure 2:**
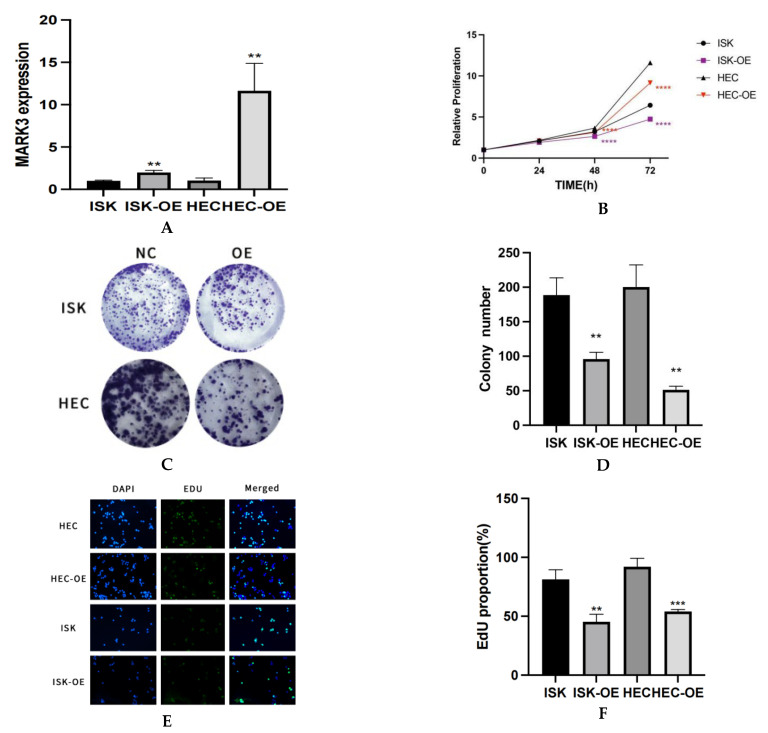
(**A**) The expression of MARK3 in different cell lines in mRNA levels via PCR. This experiment was repeated three times. Ishikawa ** *p* = 0.0027, HEC-1B ** *p* = 0.0048. (**B**) Analysis about CCK-8 Illustrating the Anti-Proliferative Effects of MARK3. The graph depicts the trends in viability, emphasizing the inhibitory impact of overexpressed MARK3 on cell proliferation in the HEC-1B and Ishikawa cell lines. This experiment was repeated three times. Ishikawa **** *p* < 0.0001, HEC-1B **** *p* < 0.0001. (**C**) The number of colonies formed by Ishikawa and HEC-1B cells overexpressing are significantly reduced compared to their respective non-overexpressing controls. (**D**) A comparative analysis chart from three repeats of colony formation between Ishikawa (** *p* = 0.0039) and HEC-1B (** *p* = 0.0014) cell lines reveals a marked decrease in colony count following overexpression, indicating a potential inhibitory effect on cellular proliferation. (**E**) Assay of Cell Proliferation and EdU Incorporation The cell proliferation in the control group, ISK-OE, and HEC-OE groups is illustrated, with merged pictures demonstrating diminished proliferative potential in the over expression groups relative to controls. Magnification: 200×. (**F**) The percentage of EdU-positive cells is markedly greater in the control group compared to the over expression groups, indicating a reduced proliferative response in those with over expression group. This experiment was repeated three times. Ishikawa ** *p* = 0.0038, HEC-1B *** *p* = 0.0009.(**, *p* < 0.01, ***, *p* < 0.001, ****, *p* < 0.0001).

RT-qPCR analysis demonstrated a notable elevation in MARK3 mRNA expression in overexpressed endometrial cancer cell lines relative to normal endometrial cancer cell lines. Figure 2A visualization effectively illustrates the differential expression of MARK3 among the various different cell lines. The increased expression levels, which are statistically significant (*p* < 0.05), indicate that MARK3 is over expressed in endometrial cancer cell lines.

The CCK-8 assay provided evidence for MARK3 overexpression and inhibited the proliferation of endometrial cancer cells, demonstrating a significant decrease in the proliferation rate of MARK3-overexpressing cells relative to control groups. Figure 2B illustrates viability trends, indicating that the proliferation of Ishikawa and HEC-1B cell lines increased over time, represented in black. In contrast, MARK3-overexpressing cells (Ishikawa OE and HEC-OE) displayed minimal changes in proliferation, as shown in red, respectively. The data indicate that MARK3 overexpression inhibits cellular proliferation.

A colony formation assay was conducted to evaluate MARK3 overexpression. Cells were cultured at low densities for 14 days to facilitate colony formation. Figure 2C,D exhibited Ishikawa cells overexpressing MARK3 reduced colony formation relative to control cells, with a *p*-value of 0.0039, indicating a potential deleterious impact on cell proliferation and survival. Similar patterns were also seen in HEC-1B cell lines, indicating that MARK3 may have a role in growth regulation (*p*-value 0.0014; Figure 2A,B).

The EDU assay corroborated the decrease in proliferation noted in the CCK-8 assay. Figure 2E,F presents images of EDU-positive (green) and DAPI-stained (blue) cells, revealing that the MARK3-overexpressing groups exhibited a significant reduction in EDU incorporation, which suggests diminished proliferative activity. Statistical analysis indicated *p*-values of 0.0038 for Ishikawa cells and 0.0009 for HEC cells, confirming the inhibitory effects of MARK3 on cellular proliferation.

### 3.3. MARK3 Could Suppresses Migration of Endometrial Cancer Cell Lines

**Figure 3:**
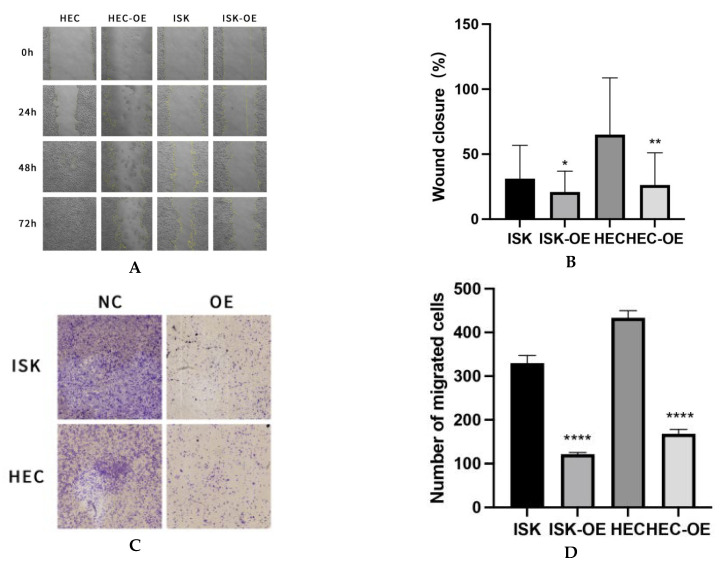
(**A**) Wound Healing Assay: The wound healing rate is significantly decreased in HEC-OE and ISK-OE cells relative to the control group. Magnification: 100×. (**B**) The quantification of wound closure reveals a significant decrease in the percentage of closure in HEC-OE and ISK-OE cells compared to the control group, signifying compromised migratory ability. This experiment was repeated three times. Ishikawa * *p* = 0.0237, HEC-1B ** *p* = 0.0040. (**C**) Illustrates the reduced migration of HEC-OE and ISK-OE in comparison to the control group in Transwell. Magnification: 200×. (**D**) Demonstrate that the migrating cell ratio is greater in the control group than in the HEC-OE and ISK-OE groups. This experiment was repeated three times. Ishikawa **** *p* < 0.0001, HEC-1B **** *p* < 0.0001.(*, *p* < 0.05, **, *p* < 0.01, ****, *p* < 0.0001).

A scratch wound healing assay was conducted at four time points (0, 24, 48, and 72 h) to evaluate the effect of MARK3 on cellular migration. Cells overexpressing MARK3, specifically Ishikawa and HEC-1B, demonstrated markedly slower wound closure rates in comparison to control cells, achieving statistical significance (*p* = 0.0237 for Ishikawa cells and *p* = 0.0040 for HEC-1B cells). Figure 3A,B illustrate the impaired migration of MARK3-overexpressing cells, indicating overexpressed MARK3’s potential role in inhibiting cell motility and migration.

The inhibitory effect of MARK3 on migration was validated through Transwell migration assays, which demonstrated a significantly reduced number of cells migrating through the membrane in the MARK3-overexpressing groups. Figure 3C,D demonstrate a significant reduction in cell migration, with *p*-values below 0.0001 for both Ishikawa and HEC-1B cell lines, highlighting the role of MARK3 in suppressing metastatic potential.

These findings indicate that MARK3 is essential for the regulation of cell proliferation and migration in endometrial cancer cells. The observed effects of reduced proliferation and decreased migration indicate that MARK3 may serve as a potential therapeutic target for endometrial cancer.

### 3.4. MARK3 Inhibits Endometrial Cancer Cell Proliferation via the PI3K/AKT/mTOR Pathway

**Figure 4:**
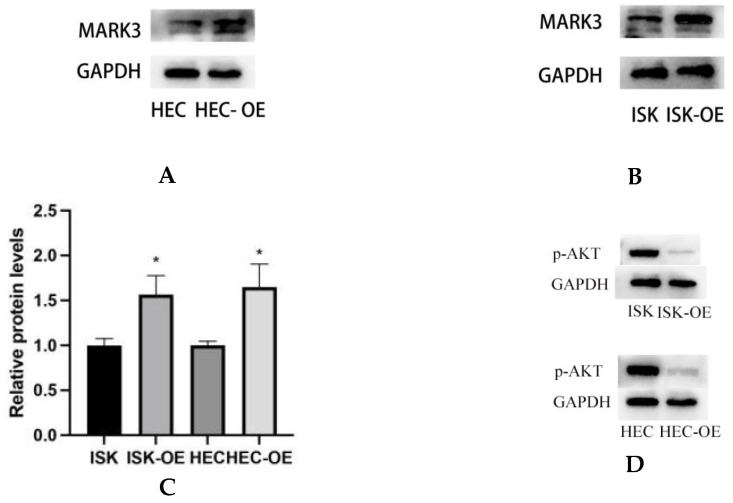

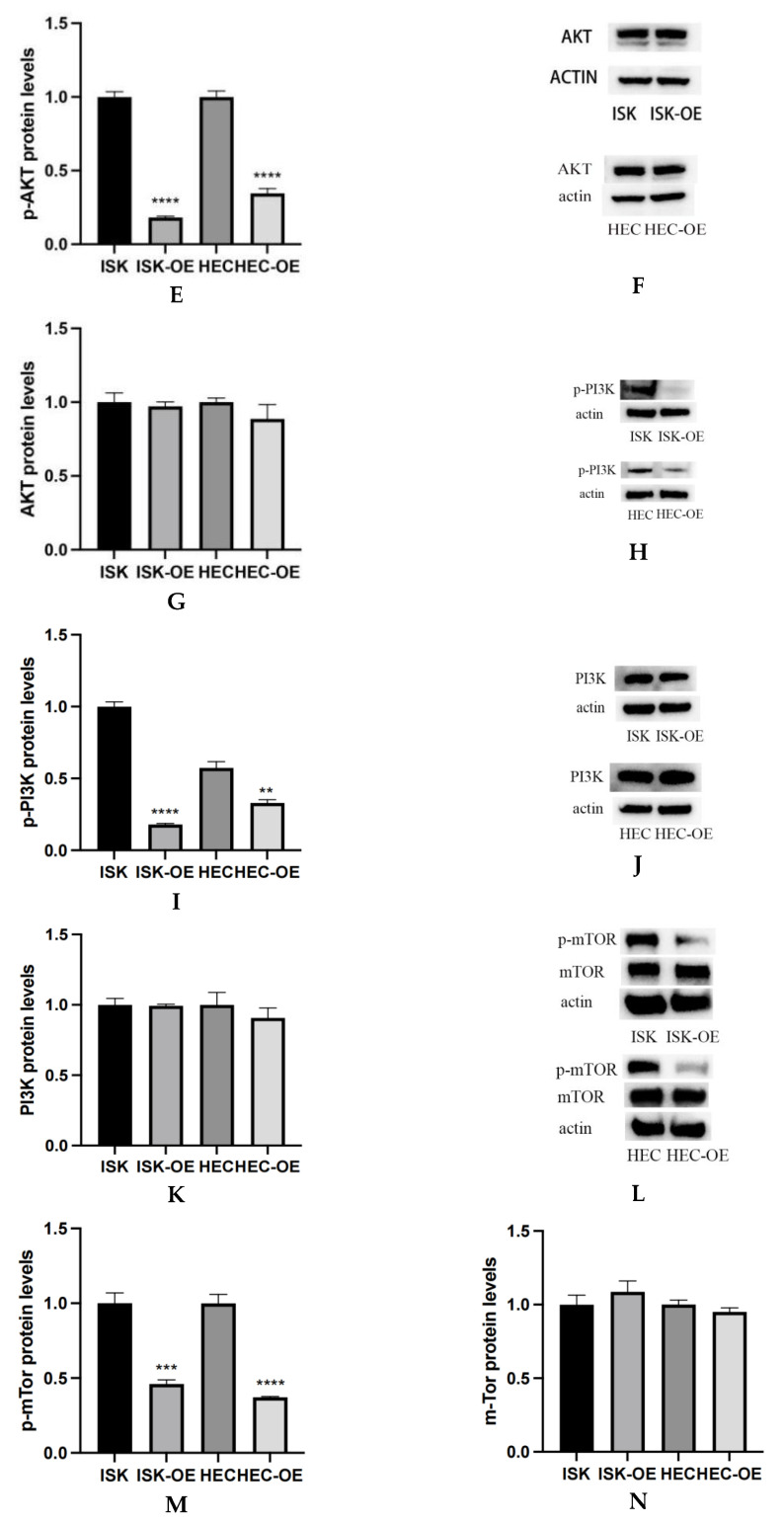

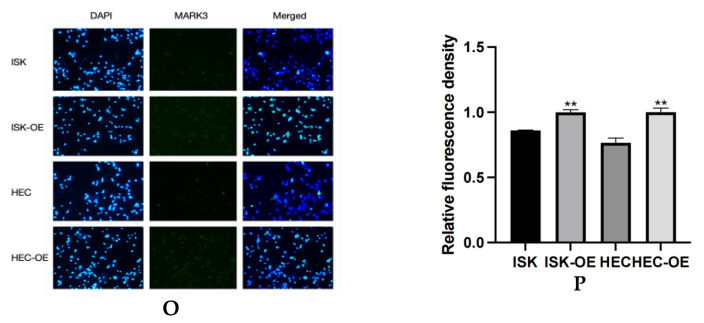
(**A**,**B**) Expression of MARK3 which compare with Ishikawa,HEC-1B and their over expression cell lines. (**C**) A comparison of relative protein levels between the control and over expression groups demonstrates considerably elevated expression of MARK3 in the over expression group. Grayscale analysis chart from three repeats. Ishikawa * *p* = 0.0121, HEC-1B * *p* = 0.0124. (**D**) Western blot showing the expression of p-AKT protein levels was decreased in MARK3 overexpression cell lines than control cell lines. (**E**) Grayscale analysis chart from three repeats. Ishikawa **** *p* < 0.0001, HEC-1B **** *p* < 0.0001. (**F**) Western blot showing the expression of AKT protein was not significantly different between MARK3 overexpression cells than control cells. (**G**) Grayscale analysis chart from three repeats. Ishikawa *p* = 0.5189, HEC-1B *p* = 0.1239. (**H**) Western blot showing the expression of p-PI3K protein levels was decreased in MARK3 over-expression cell lines than control cell lines. (**I**) Grayscale analysis chart from three repeats. Ishikawa **** *p* < 0.0001, HEC-1B ** *p* = 0.0011. (**J**) Western blot showing the expression of PI3K protein was not significantly different between MARK3 over-expression cells and control cells. (**K**) Grayscale analysis chart from three repeats. Ishikawa *p* = 0.8162, HEC-1B *p* = 0.2297. (**L**) Western blot showing the expression of p-mTOR protein was decreased in over-expression cell lines than control cell lines. And the mTOR protein was not significantly different. The uncropped blots are shown in the Supplemental Materials S3. (**M**) Grayscale analysis chart from three repeats. Ishikawa *** *p* = 0.0002,HEC-1B **** *p* < 0.0001. (**N**) Grayscale analysis chart from three repeats. Ishikawa *p* = 0.1974,HEC-1B *p* = 0.1112. (**O**) Illustrate the MARK3 expression in over expression group and control group and show expression is high in over expressed cells. (**P**) Analysis the relative fluorescence density in over expression group and control group. This experiment was repeated three times. Ishikawa *p* = 0.0022, HEC-1B *p* = 0.0081 (*, *p* < 0.05, **, *p* < 0.01, ***, *p* < 0.001, ****, *p* < 0.0001).

Western blot analysis was conducted to assess the impact of MARK3 overexpression on critical regulatory proteins in endometrial cancer cell lines, Ishikawa and HEC-1B. Figure 4A–C demonstrate that MARK3 expression was significantly increased in cells overexpressing MARK3 (HEC-OE and Ishikawa-OE) relative to control groups. Quantitative analysis indicated *p*-values of 0.0121 and 0.0124 for HEC-OE and Ishikawa-OE, respectively, thereby confirming the overexpression of MARK3.

The activation of cell proliferation and migration pathways was examined through the analysis of AKT, PI3K, and mTOR expression and its phosphorylated variant (p-AKT, p-PI3K, and p-mTOR). AKT is a crucial protein that plays a significant role in cell survival and proliferation, whereas p-AKT functions as a marker for pathway activation. Figure 4F,G indicate that total AKT expression did not exhibit a significant difference between overexpressing and control cells (HEC *p* = 0.1239, Ishikawa *p* = 0.5189). Analysis of p-AKT levels (Figure 4D,E) revealed a significant decrease in phosphorylated AKT in both HEC-OE and Ishikawa-OE cells (*p* < 0.0001); both p-PI3K and p-mTOR were significantly downregulated in the MARK3 overexpression groups(*p* < 0.05) (Figure 4H,I,L,M),while the levels of PI3K and mTOR remained largely unchanged (Figure 4J,K,L,N). This indicates that MARK3 overexpression may inhibit the activation of the PI3K/AKT/mTOR signaling pathway, which is associated with cell survival, tumor progression, and resistance to therapeutic interventions.

Immunofluorescence assays were performed to examine the subcellular localization of MARK3 in transfected cells. Figure 4O,P display the distribution of MARK3 within the cytoplasm and nucleus, with merged images indicating elevated expression in cells with overexpression. The statistical analysis indicated *p*-values of 0.0022 for Ishikawa cells and 0.0081 for HEC cells, confirming the overexpression of MARK3.

The findings indicate that MARK3 overexpression significantly affects critical cellular processes, including survival and migration in endometrial cancer cells. MARK3 may inhibit cancer cell survival through the modulation of PI3K/AKT/mTOR pathway, thereby inhibiting tumor progression. The findings indicate MARK3 as a potential therapeutic target for strategies designed to disrupt the survival mechanisms of endometrial cancer cells.

## 4. Discussion

Endometrial cancer (EC) represents the most prevalent gynecologic malignancy in developed nations, with its incidence linked to factors including aging and lifestyle modifications, notably obesity and hormonal influences. Prognostic outcomes remain challenging to predict, especially for cases identified at advanced stages, despite advancements in detection methods. The development of novel biomarkers is essential for enhancing disease management [11]. This research investigates the functional role of microtubule affinity-regulating kinase 3 (MARK3) in endometrial cancer for the first time. Prior studies have demonstrated the prognostic and functional significance of MARK3 in various cancers, including breast, ovarian, colorectal, and cervical cancers [12,13,14], but its role in endometrial cancer has remained unexplored until now.

Our findings demonstrate that MARK3 plays a significant role in regulating cellular processes critical for tumor progression, such as proliferation, apoptosis, and migration. This suggests that MARK3 may serve as a prognostic biomarker and therapeutic target in endometrial cancer. RT-qPCR analysis demonstrated elevated MARK3 expression in overexpression MARK3 endometrial cancer cells compared to control groups. The colony formation assay results demonstrated that MARK3 overexpression significantly diminished the long-term proliferative capacity of Ishikawa and HEC-1B cells, achieving *p*-values of 0.0039 and 0.0014, respectively, indicating its anti-proliferative effect. This aligns with findings in other cancers, suggesting that MARK3 is associated with reduced cellular proliferation and enhanced cell cycle regulation [15]. The CCK-8 assay demonstrated that cells overexpressing MARK3 showed a reduction in viability over time, consistent with its anti-proliferative effects.

The EDU assay confirmed reduced proliferation rates in MARK3 overexpressing cells, consistent with results from the CCK-8 assay, showing *p*-values of 0.0038 and 0.0009 for Ishikawa and HEC-1B cells, respectively. This supports the role of MARK3 in inhibiting endometrial cancer cell proliferation, possibly through mechanisms of cell cycle arrest, a feature observed in other cancers like ovarian and colorectal cancer. MARK3 downregulation is linked to cancer cell survival and chemoresistance, similar to findings in colorectal and breast cancers [16]. The results emphasize the influence of MARK3 on endometrial cancer.

Migration assays, including scratch wound healing and Transwell assays, indicated that MARK3 plays a role in limiting cell motility. MARK3 overexpressing Ishikawa and HEC-1B cells exhibited significantly lower wound closure rates and reduced migration through the Transwell membrane, with *p*-values < 0.0001 for both assays. The results corroborate previous studies suggesting that suppresses metastatic behavior, particularly in cancer [13,17].

The Western blot analysis demonstrated that the decreased levels of phosphorylated AKT, PI3K, and mTOR (p-AKT, p-PI3K, and p-mTOR) in MARK3 overexpressing cells indicate that MARK3 may influence downstream survival signaling pathways, thereby impacting tumor behavior through modulation of the PI3K/AKT/mTOR pathway. Prior research has established the regulation of AKT and apoptotic proteins by MARK3, highlighting its potential significance in cancer cell survival dynamics [16,17].

The immunofluorescence assay revealed that the expression of MARK3 in overexpression cells is higher than control groups. These results indicate that MARK3 may play a role in the initial phases of endometrial carcinoma development and could be involved in tumor progression. Gene enrichment analysis indicated that MARK3 is linked to essential cancer-related pathways, such as cell cycle regulation, apoptosis, and cell migration. The pathways are essential for tumor progression, and dysregulation of these processes is frequently observed in malignancies, including EC.

The co-expression analysis yielded significant insights into the molecular networks potentially linked to MARK3 in EC. A set of genes co-expressed with MARK3 was identified, which is implicated in diverse cellular processes such as DNA repair, cell cycle regulation, and apoptosis. The findings indicate that MARK3 may engage with various signaling pathways to modulate cellular behavior in endothelial cells. Some of these co-expressed genes have been previously associated with EC, thereby reinforcing the biological significance of MARK3 in this disease context.

Our findings highlight the significant role of MARK3 in regulating essential aspects of endometrial cancer progression, such as proliferation, apoptosis, and migration. The findings from experimental assays and bioinformatics analyses strongly indicate that MARK3 may serve as a prognostic biomarker and therapeutic target in endometrial cancer. Modulating these pathways may provide a novel approach to personalized treatment strategies for patients with endometrial cancer through MARK3. This study systematically evaluates the functional role of MARK3 in endothelial cells, enhancing the understanding of its mechanistic contributions to cancer biology and offering insights into potential clinical applications.

## 5. Conclusions

This research presents new evidence regarding the function of MARK3 in endometrial cancer, showing its complex impact on cancer cell dynamics through regulation of proliferation, apoptosis, and migration. MARK3 plays an essential role in EC via PI3K/AKT/mTOR pathways, making it both a prognostic biomarker and therapeutic target. Dysregulation of MARK3 expression has been found to correlate with cancer progression and survival outcomes across numerous cancer types, such as breast, ovarian, colorectal, and endometrial. These findings enhance understanding of its mechanistic role in cancer biology while supporting personalized therapeutic strategies targeting MARK3 for endometrial cancer therapy.

Further study will be necessary to ascertain the molecular mechanisms related to MARK3’s role in endometrial cancer as well as assess its clinical importance for predicting patient outcomes and informing treatment approaches.

## Data Availability

The date presented in this study are available in this article and Supplementary Material.

## Supplementary Materials

The following supporting information can be downloaded at: https://www.mdpi.com/article/10.3390/curroncol32030157/s1, S1: corResult supplementary; S2: GO supplementary; S3: Full pictures of the Western blots in Figure 4.
