# Supplementary figures and images for: Mark3 a Prognostic Marker for the Endometrial Cancer (part 2 of 2)
Source: Curr Oncol. 2025 Mar 10;32(3):157. doi: 10.3390/curroncol32030157 (PMC11941562; doi:10.3390/curroncol32030157)

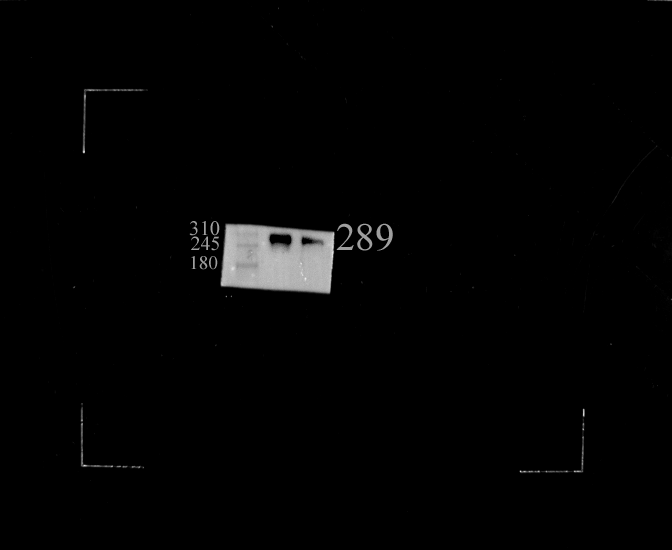

Supplement: Supplementary file 1 [file curroncol-32-00157-s001.zip › WB-supplementary S3/Figure4-L/ISK_P_MTOR _8bit_8bit_8bit_8bit.tif]

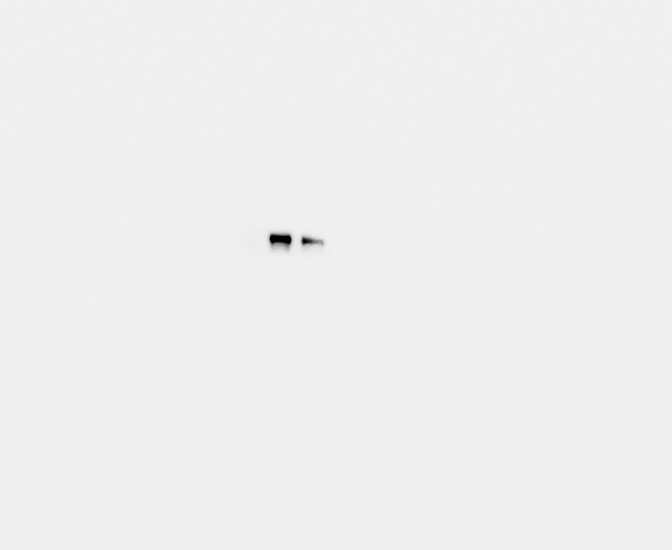

Supplement: Supplementary file 1 [file curroncol-32-00157-s001.zip › WB-supplementary S3/Figure4-L/ISK_P_MTOR_8bit_8bit_8bit.tif]

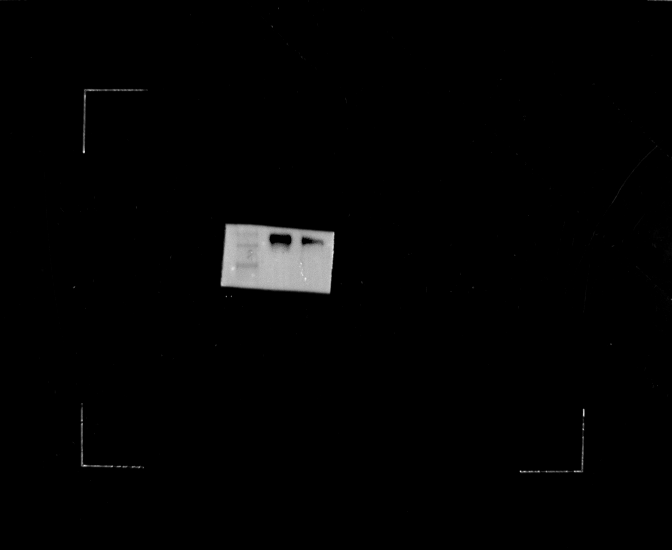

Supplement: Supplementary file 1 [file curroncol-32-00157-s001.zip › WB-supplementary S3/Figure4-L/ISK_P_MTOR_8bit_8bit_8bit_8bit.tif]

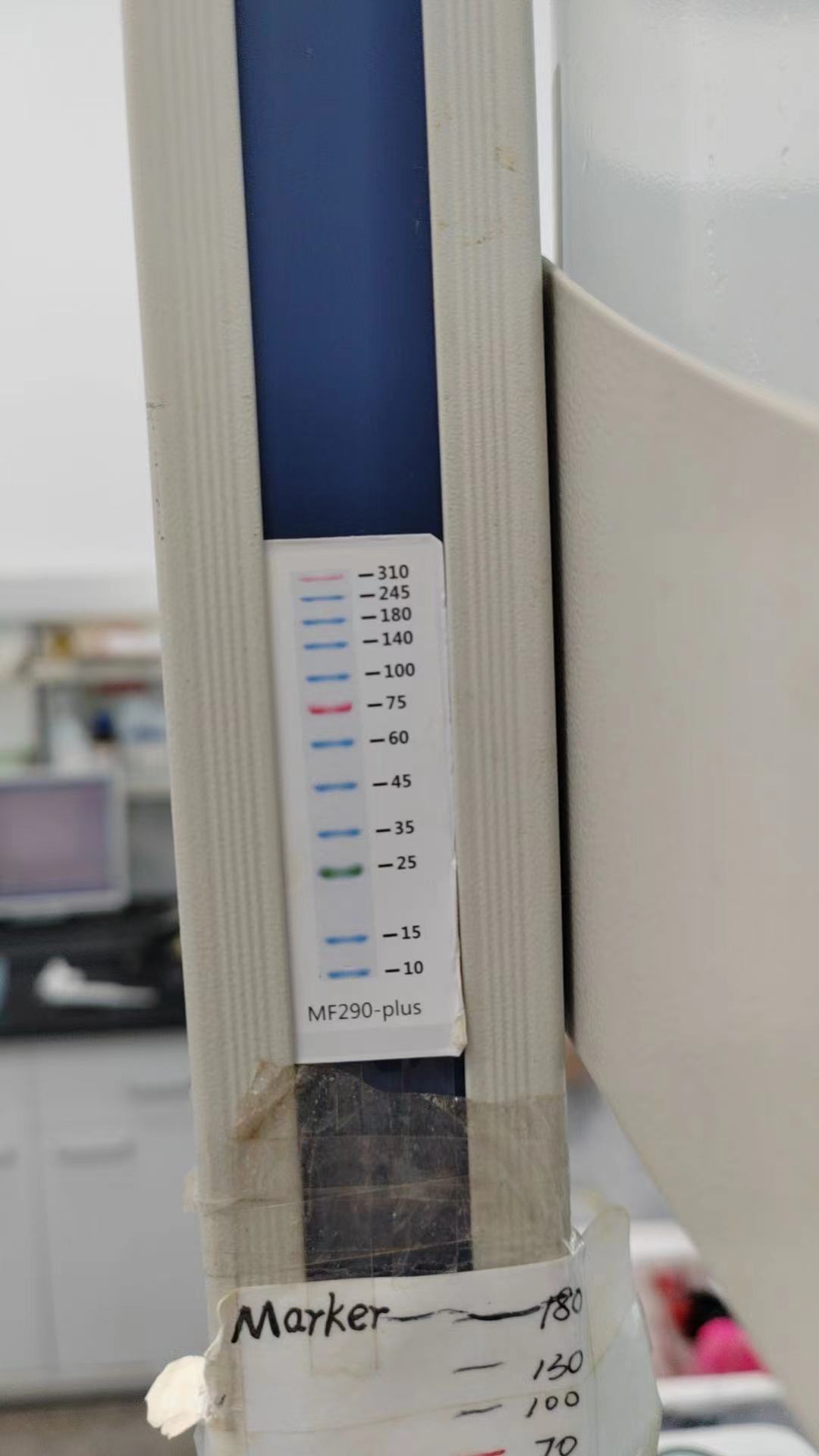

Supplement: Supplementary file 1 [file curroncol-32-00157-s001.zip › WB-supplementary S3/Figure4-L/WechatIMG465.jpg]
