# Supplementary figures and images for: Mark3 a Prognostic Marker for the Endometrial Cancer (part 1 of 2)
Source: Curr Oncol. 2025 Mar 10;32(3):157. doi: 10.3390/curroncol32030157 (PMC11941562; doi:10.3390/curroncol32030157)

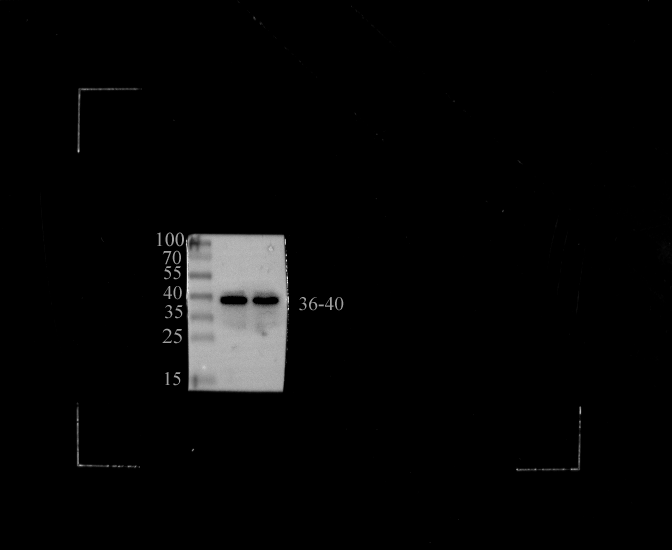

Supplement: Supplementary file 1 [file curroncol-32-00157-s001.zip › WB-supplementary S3/Figure4-A/HEC_1_GAP_8bit.png]

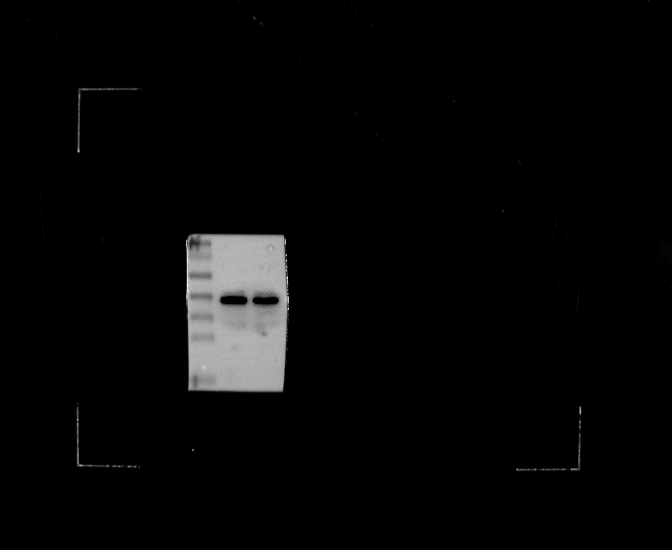

Supplement: Supplementary file 1 [file curroncol-32-00157-s001.zip › WB-supplementary S3/Figure4-A/HEC_1_GAP_8bit.tif]

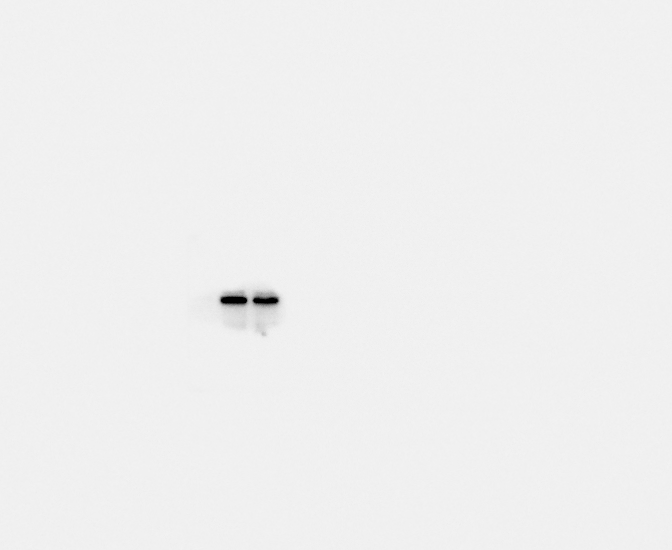

Supplement: Supplementary file 1 [file curroncol-32-00157-s001.zip › WB-supplementary S3/Figure4-A/HEC_1_GAP_8bit_8bit.tif]

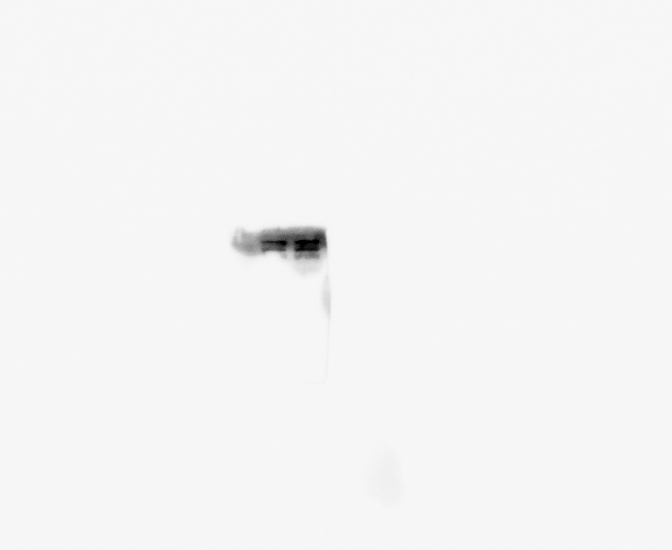

Supplement: Supplementary file 1 [file curroncol-32-00157-s001.zip › WB-supplementary S3/Figure4-A/HEC_1_MARK3_8bit.tif]

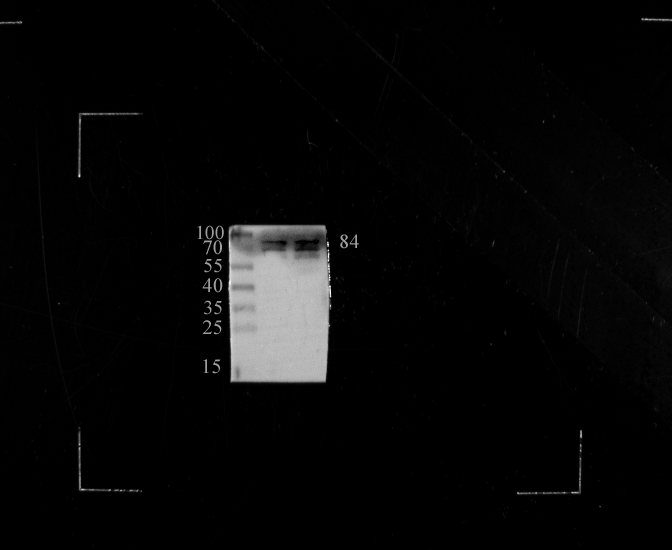

Supplement: Supplementary file 1 [file curroncol-32-00157-s001.zip › WB-supplementary S3/Figure4-A/HEC_1_MARK3_8bit_8bit.png]

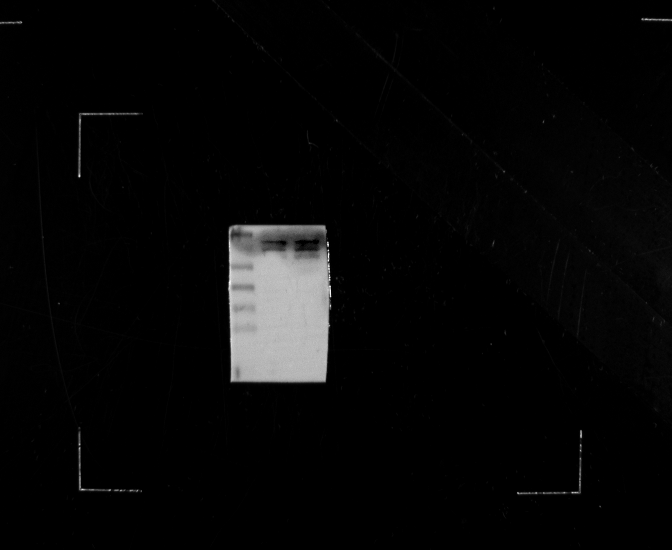

Supplement: Supplementary file 1 [file curroncol-32-00157-s001.zip › WB-supplementary S3/Figure4-A/HEC_1_MARK3_8bit_8bit.tif]

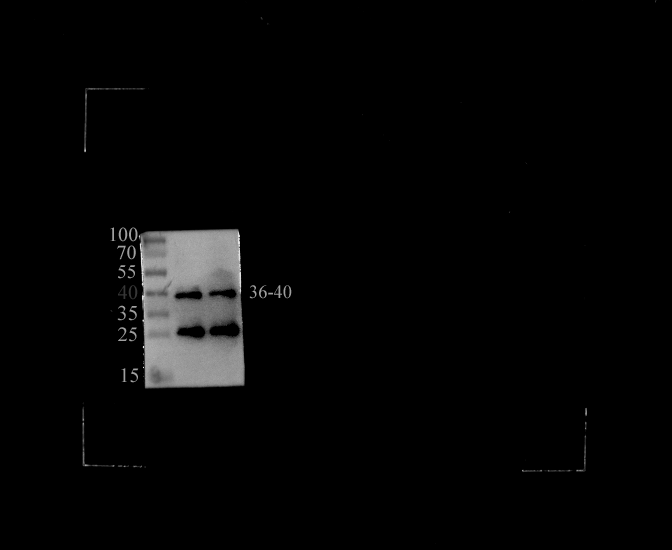

Supplement: Supplementary file 1 [file curroncol-32-00157-s001.zip › WB-supplementary S3/Figure4-B/ISK_3_GAP_8bit_8bit_8bit.png]

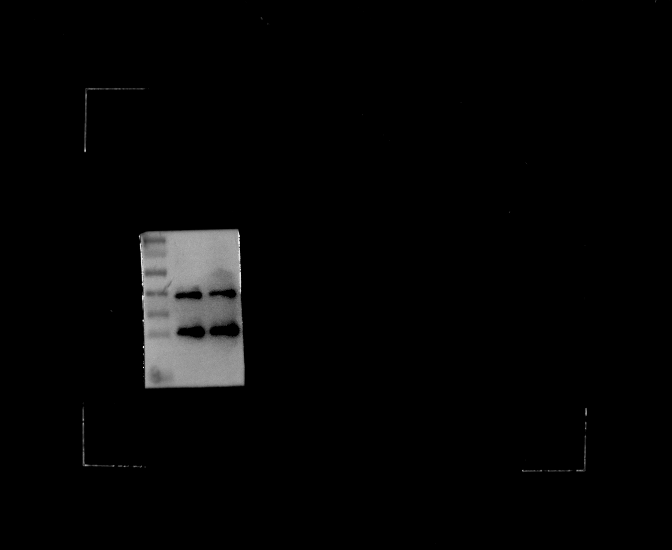

Supplement: Supplementary file 1 [file curroncol-32-00157-s001.zip › WB-supplementary S3/Figure4-B/ISK_3_GAP_8bit_8bit_8bit.tif]

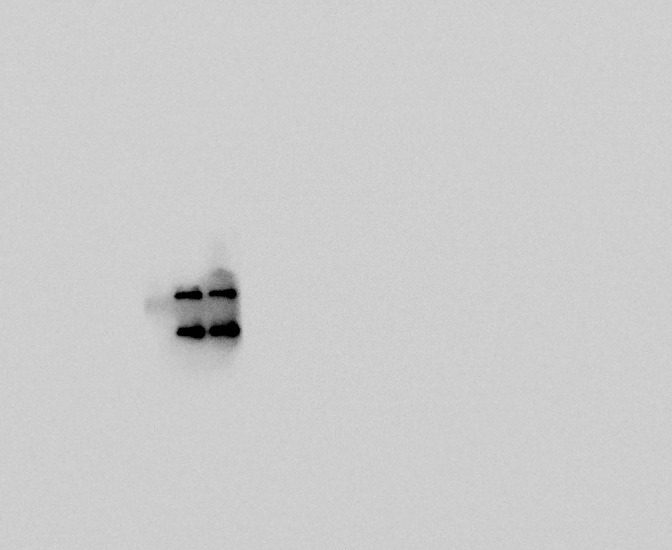

Supplement: Supplementary file 1 [file curroncol-32-00157-s001.zip › WB-supplementary S3/Figure4-B/ISK_3_GAP_8bit_8bit_8bit_8bit.tif]

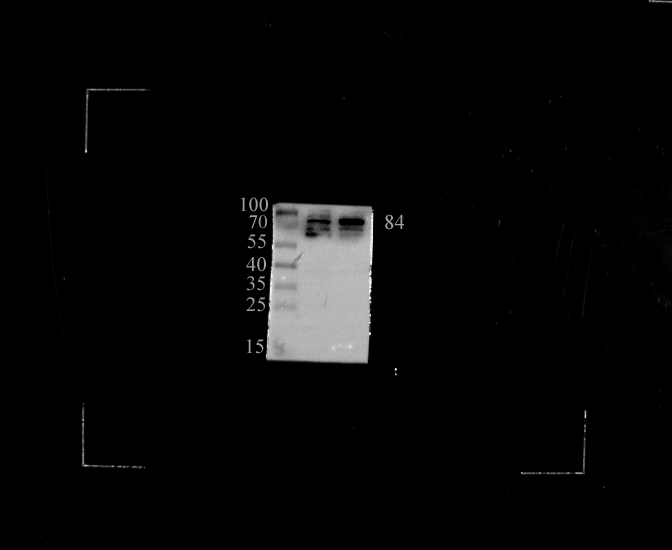

Supplement: Supplementary file 1 [file curroncol-32-00157-s001.zip › WB-supplementary S3/Figure4-B/ISK_3_MARK3_8bit.png]

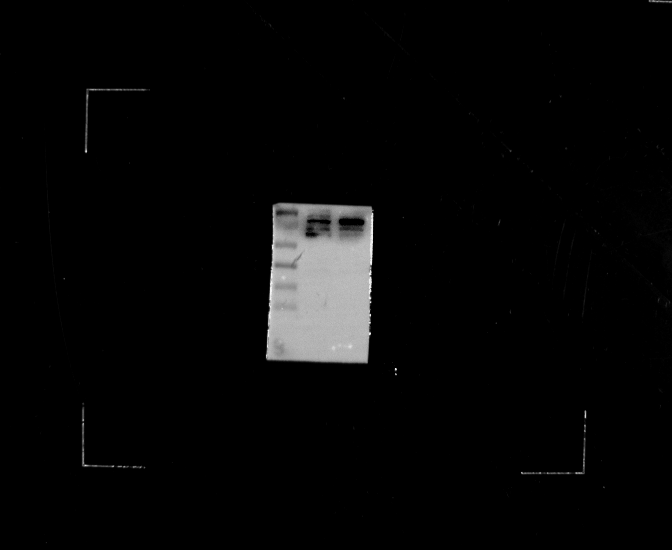

Supplement: Supplementary file 1 [file curroncol-32-00157-s001.zip › WB-supplementary S3/Figure4-B/ISK_3_MARK3_8bit.tif]

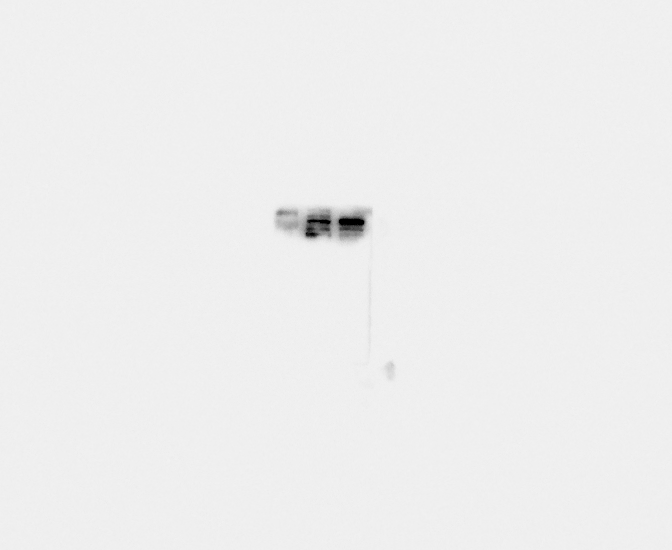

Supplement: Supplementary file 1 [file curroncol-32-00157-s001.zip › WB-supplementary S3/Figure4-B/ISK_3_MARK3_8bit_8bit.tif]

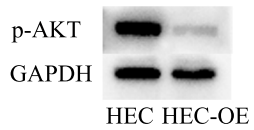

Supplement: Supplementary file 1 [file curroncol-32-00157-s001.zip › WB-supplementary S3/Figure4-D/HEC-p-AKT.pdf]

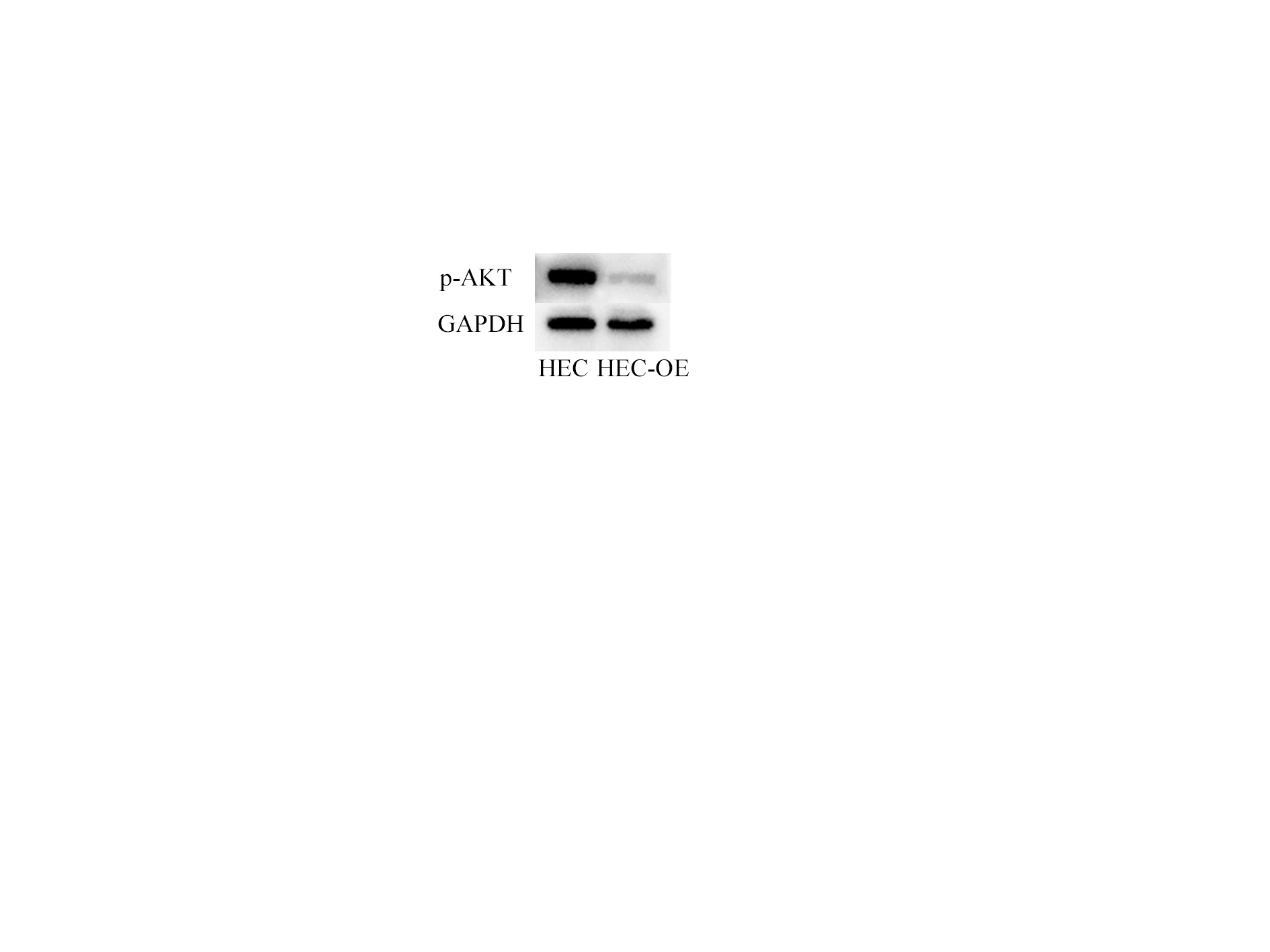

Supplement: Supplementary file 1 [file curroncol-32-00157-s001.zip › WB-supplementary S3/Figure4-D/HEC-p-AKT.png]

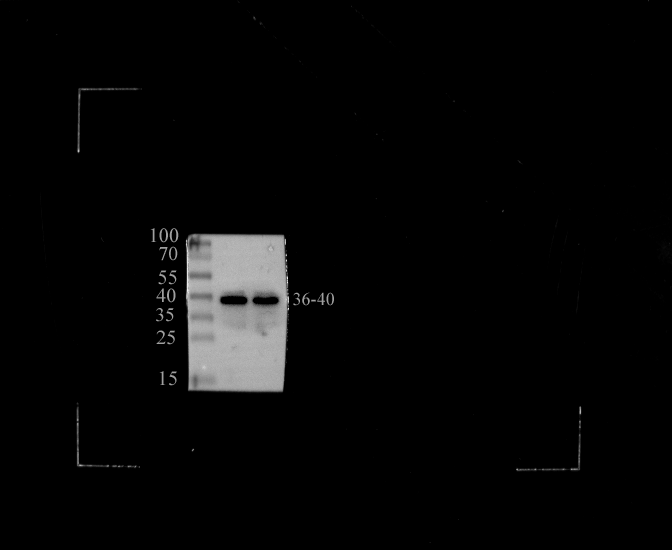

Supplement: Supplementary file 1 [file curroncol-32-00157-s001.zip › WB-supplementary S3/Figure4-D/HEC_1_GAP_8bit.png]

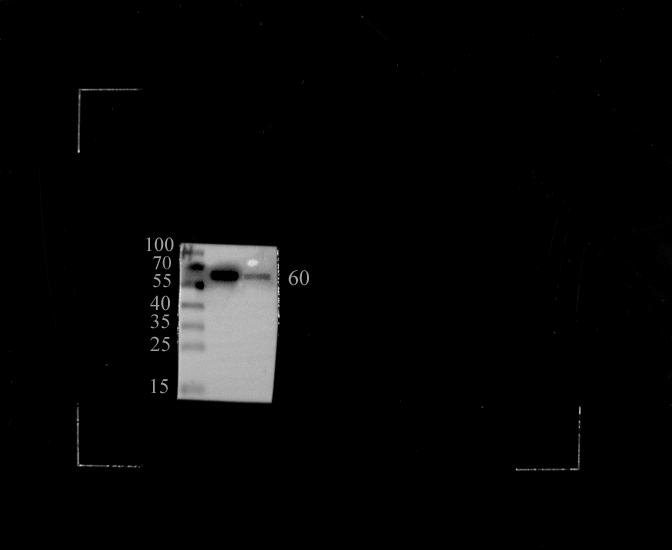

Supplement: Supplementary file 1 [file curroncol-32-00157-s001.zip › WB-supplementary S3/Figure4-D/HEC_1_P_AKT_8bit_8bit.png]

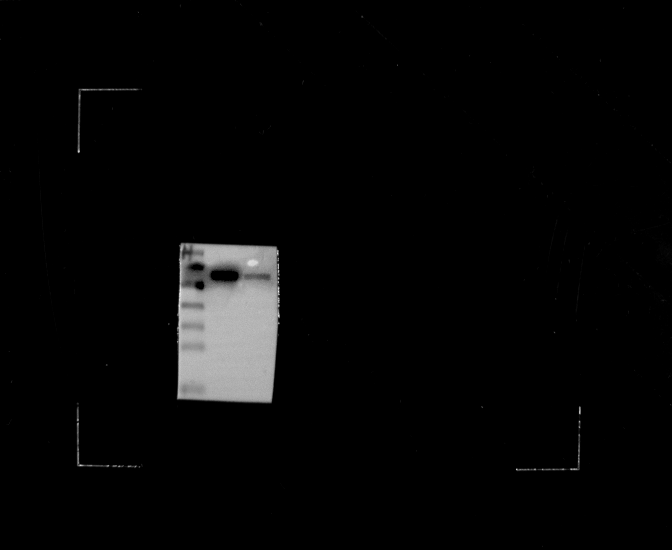

Supplement: Supplementary file 1 [file curroncol-32-00157-s001.zip › WB-supplementary S3/Figure4-D/HEC_1_P_AKT_8bit_8bit.tif]

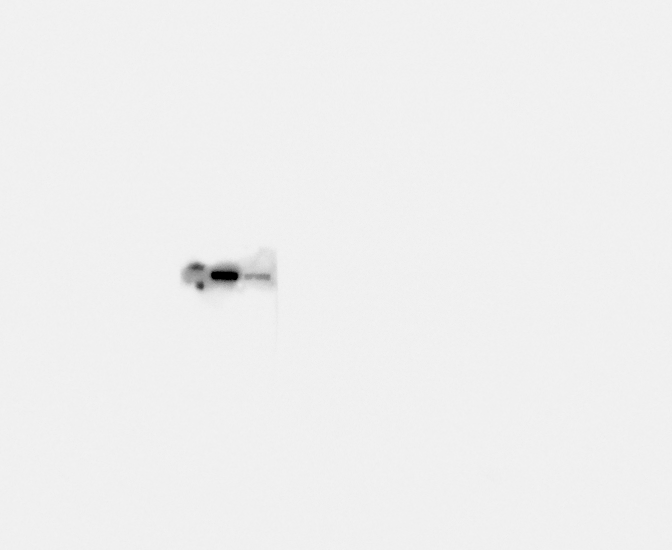

Supplement: Supplementary file 1 [file curroncol-32-00157-s001.zip › WB-supplementary S3/Figure4-D/HEC_1_P_AKT_8bit_8bit_8bit.tif]

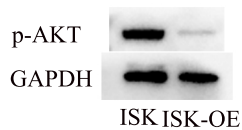

Supplement: Supplementary file 1 [file curroncol-32-00157-s001.zip › WB-supplementary S3/Figure4-D/ISK-p-AKT.pdf]

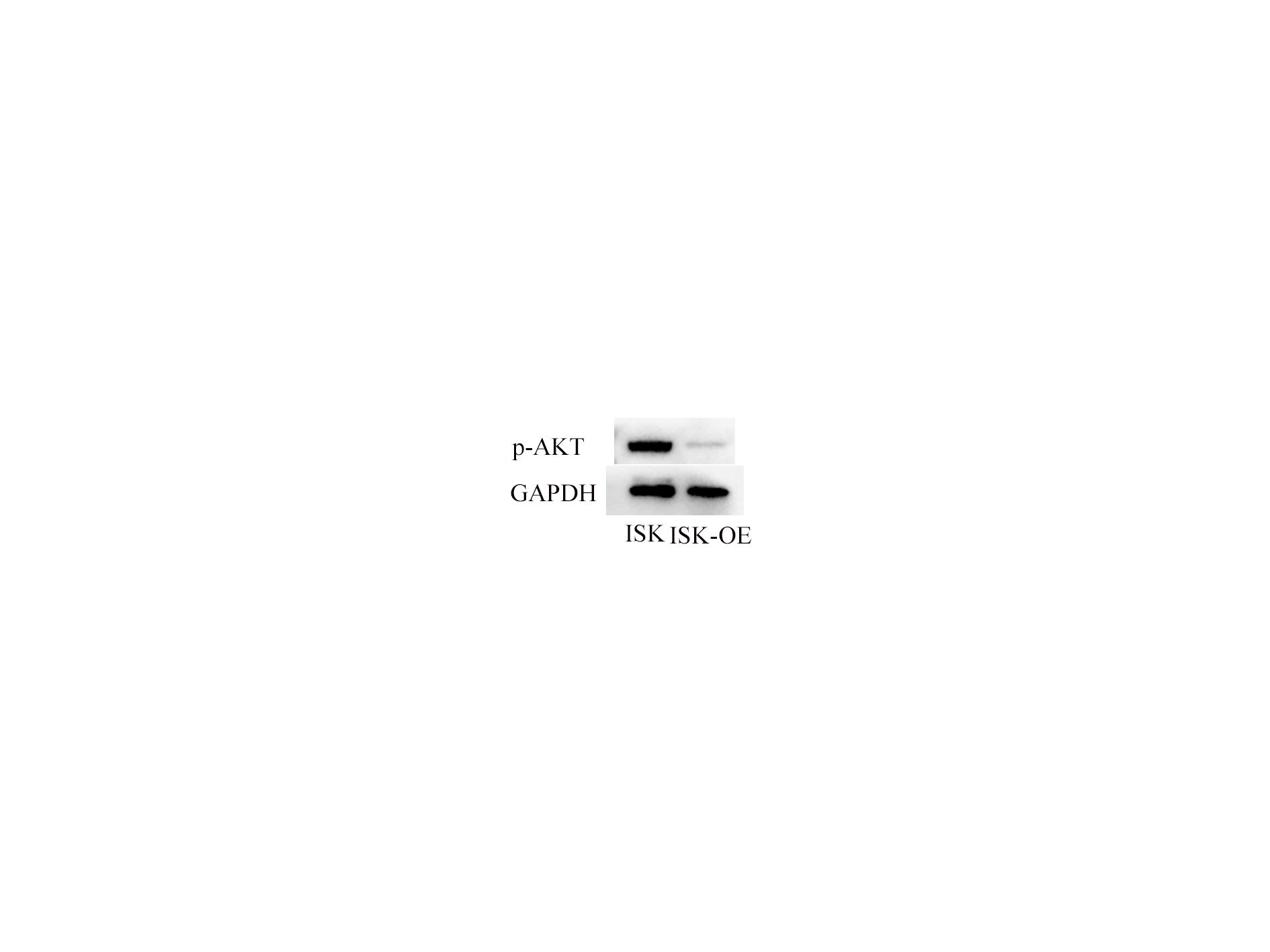

Supplement: Supplementary file 1 [file curroncol-32-00157-s001.zip › WB-supplementary S3/Figure4-D/ISK-p-AKT.png]

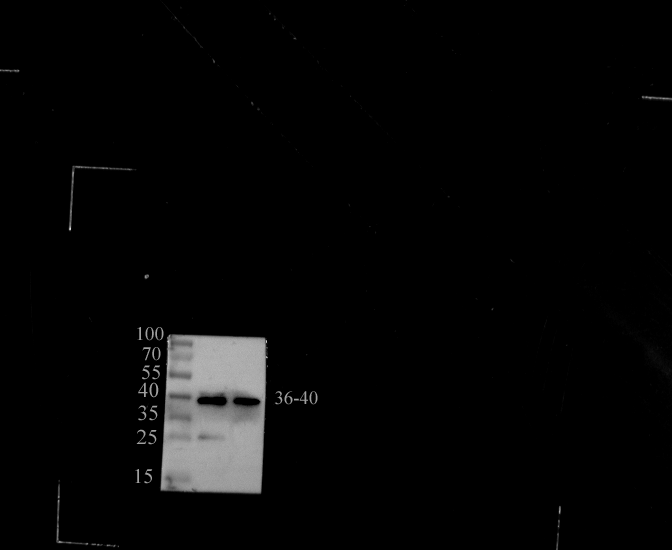

Supplement: Supplementary file 1 [file curroncol-32-00157-s001.zip › WB-supplementary S3/Figure4-D/ISK_2_GAP_8bit.png]

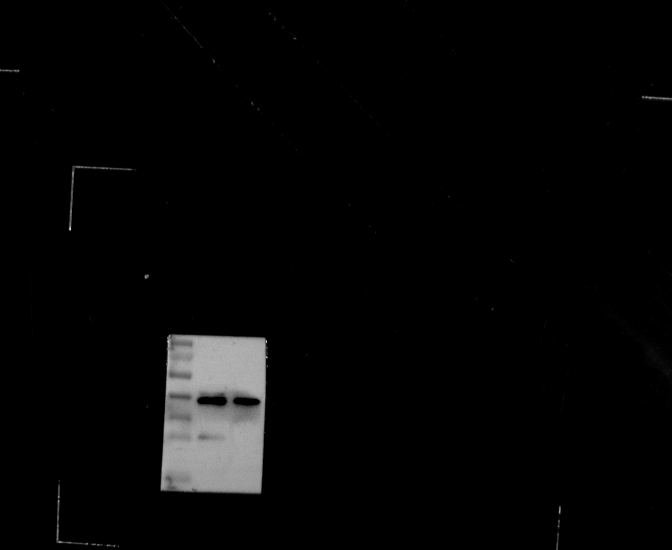

Supplement: Supplementary file 1 [file curroncol-32-00157-s001.zip › WB-supplementary S3/Figure4-D/ISK_2_GAP_8bit.tif]

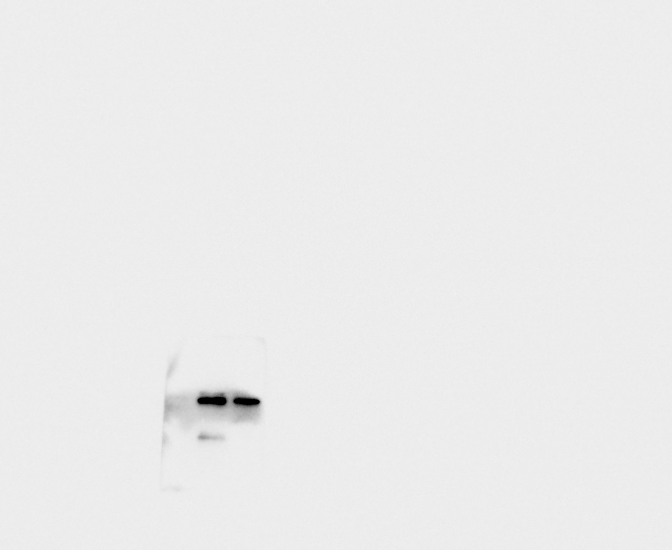

Supplement: Supplementary file 1 [file curroncol-32-00157-s001.zip › WB-supplementary S3/Figure4-D/ISK_2_GAP_8bit_8bit.tif]

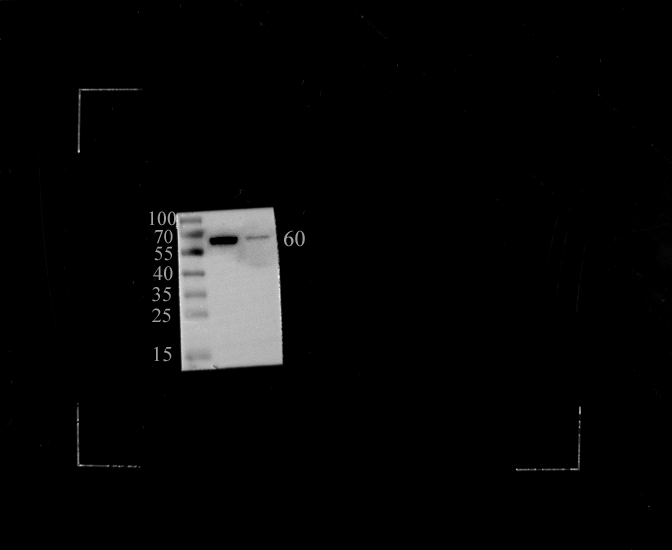

Supplement: Supplementary file 1 [file curroncol-32-00157-s001.zip › WB-supplementary S3/Figure4-D/ISK_2_P_AKT_8bit.png]

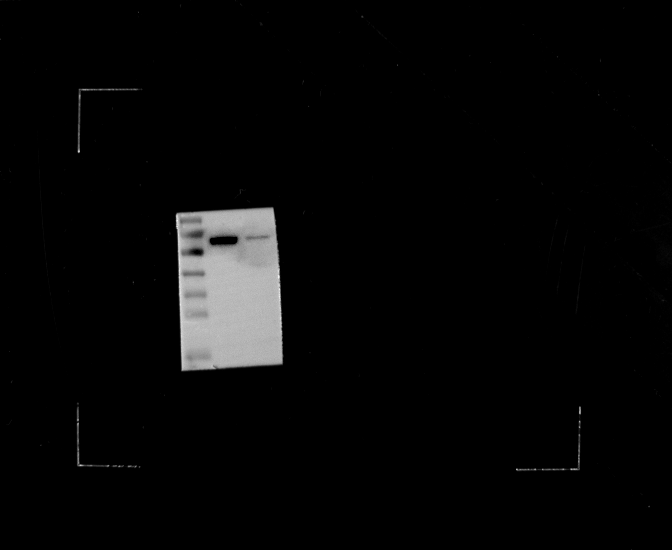

Supplement: Supplementary file 1 [file curroncol-32-00157-s001.zip › WB-supplementary S3/Figure4-D/ISK_2_P_AKT_8bit.tif]

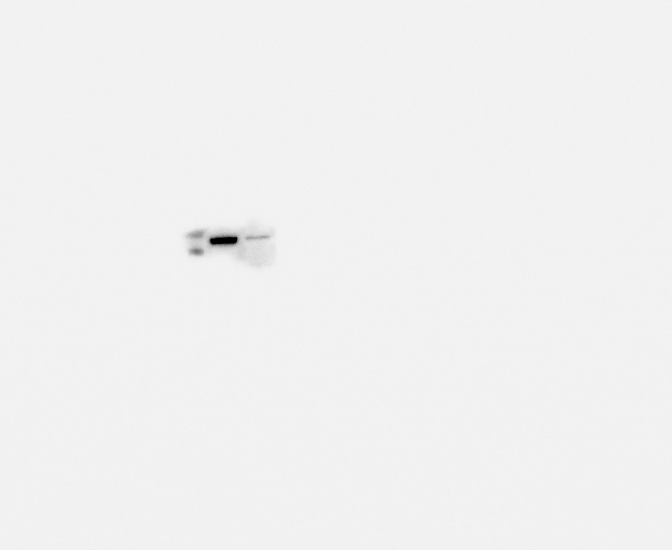

Supplement: Supplementary file 1 [file curroncol-32-00157-s001.zip › WB-supplementary S3/Figure4-D/ISK_2_P_AKT_8bit_8bit.tif]

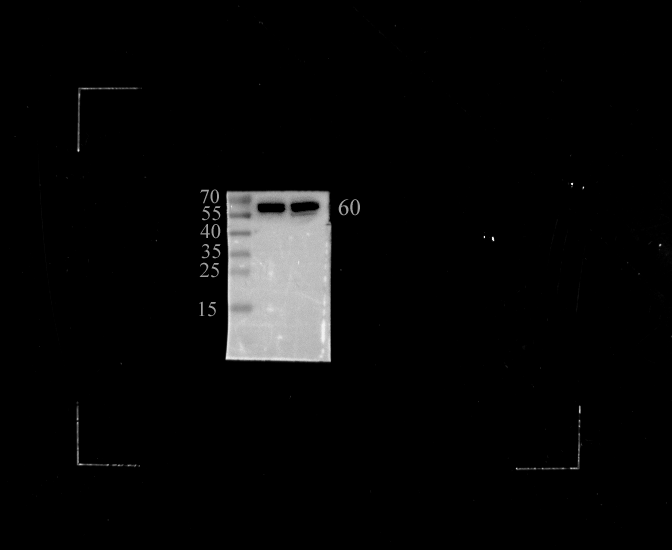

Supplement: Supplementary file 1 [file curroncol-32-00157-s001.zip › WB-supplementary S3/Figure4-F/HEC-AKT-.png]

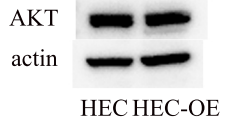

Supplement: Supplementary file 1 [file curroncol-32-00157-s001.zip › WB-supplementary S3/Figure4-F/HEC-AKT.pdf]

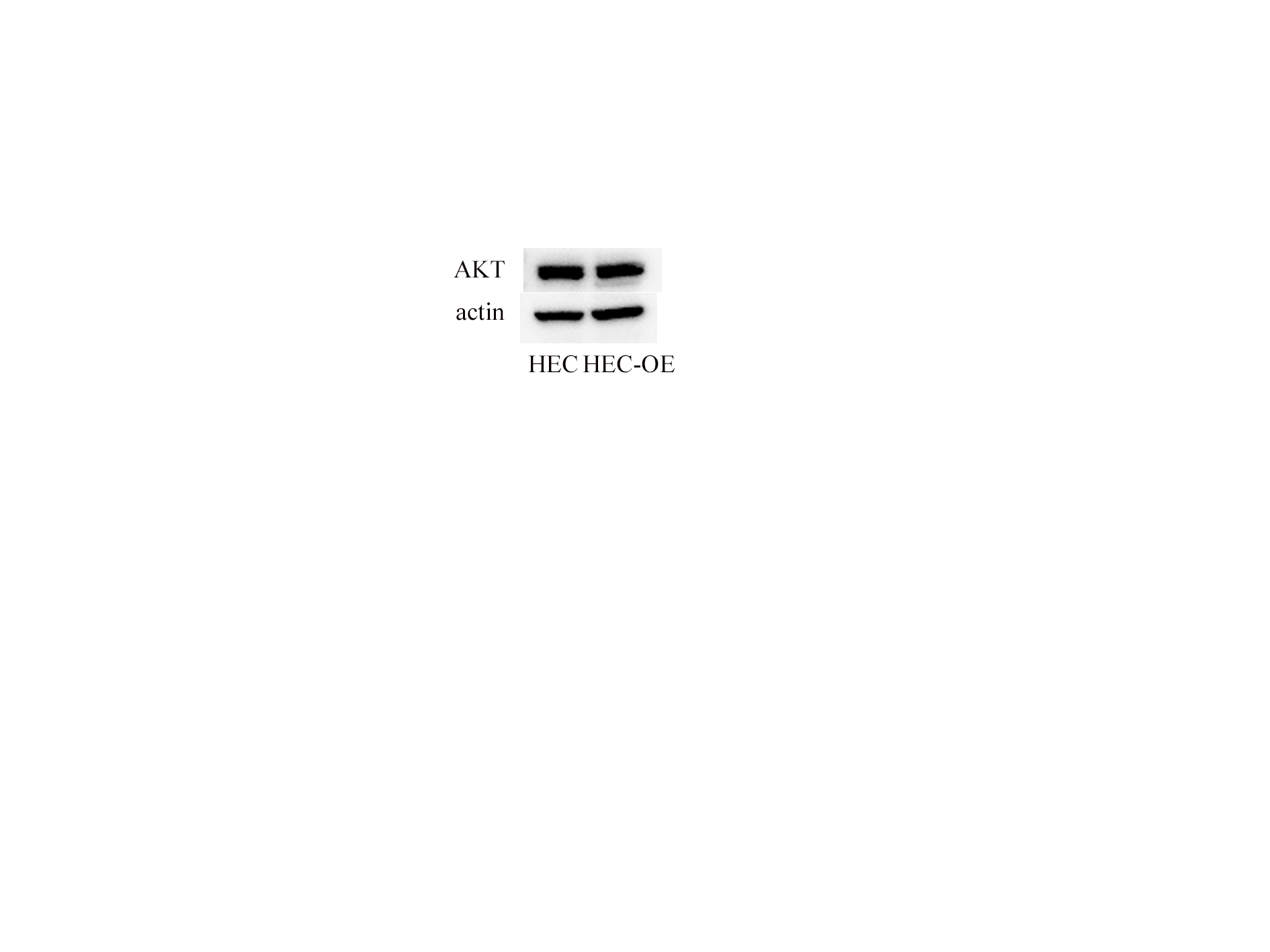

Supplement: Supplementary file 1 [file curroncol-32-00157-s001.zip › WB-supplementary S3/Figure4-F/HEC-AKT.png]

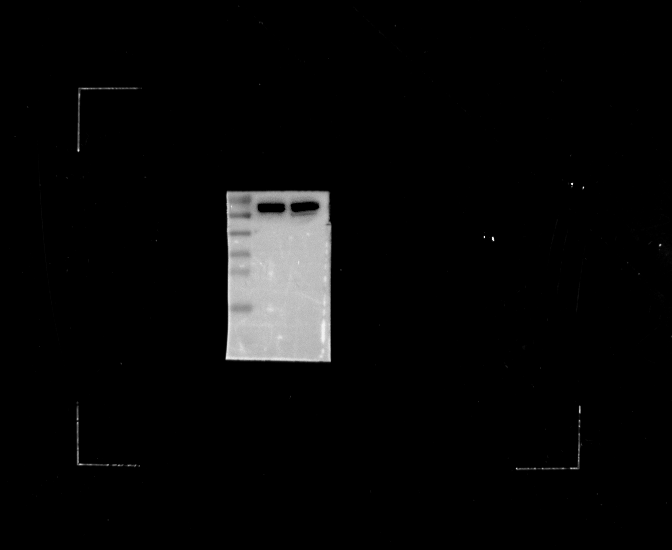

Supplement: Supplementary file 1 [file curroncol-32-00157-s001.zip › WB-supplementary S3/Figure4-F/HEC-AKT.tif]

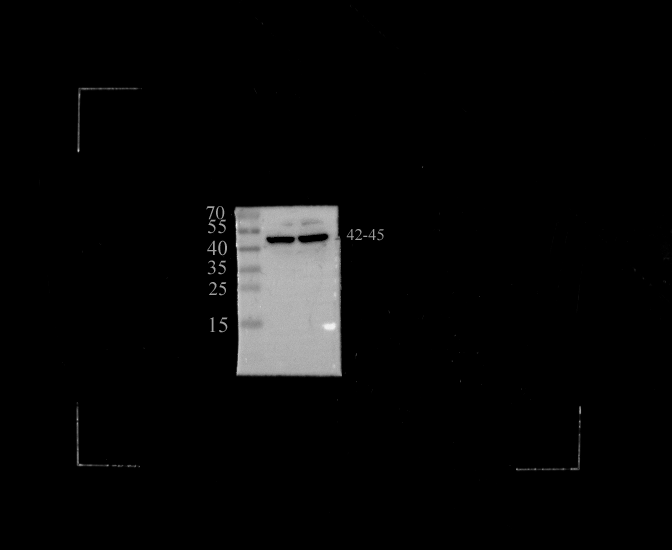

Supplement: Supplementary file 1 [file curroncol-32-00157-s001.zip › WB-supplementary S3/Figure4-F/HEC_ACTIN_8bit.png]

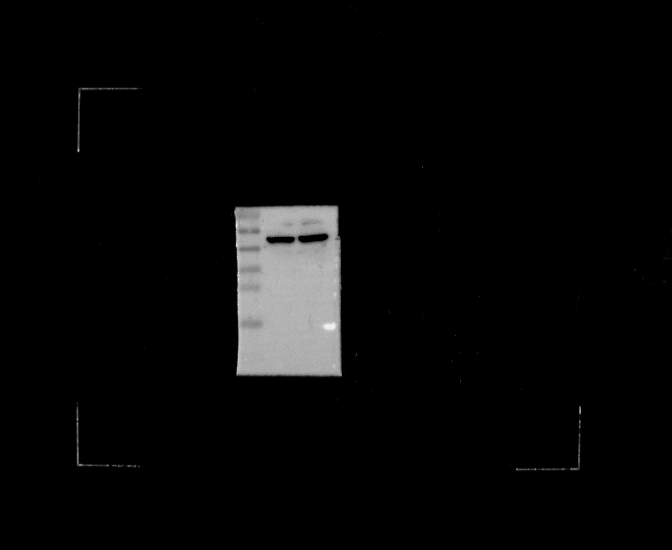

Supplement: Supplementary file 1 [file curroncol-32-00157-s001.zip › WB-supplementary S3/Figure4-F/HEC_ACTIN_8bit.tif]

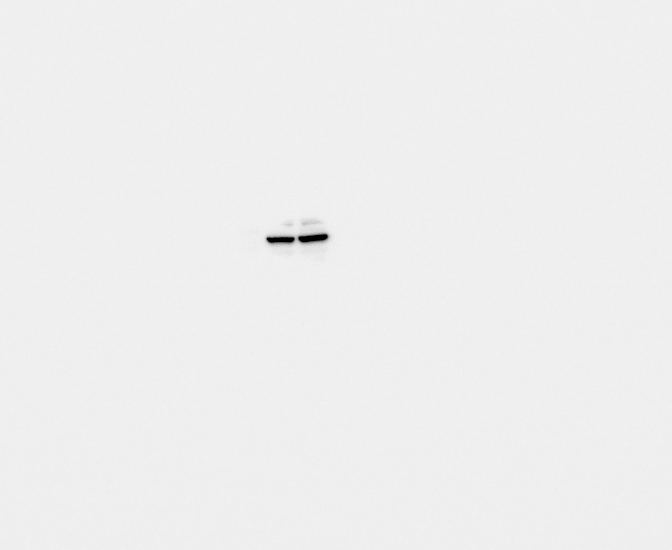

Supplement: Supplementary file 1 [file curroncol-32-00157-s001.zip › WB-supplementary S3/Figure4-F/HEC_ACTIN_8bit_8bit.tif]

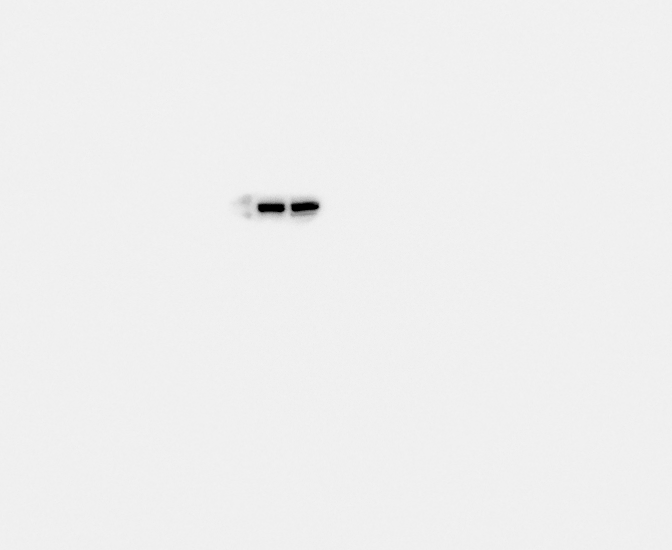

Supplement: Supplementary file 1 [file curroncol-32-00157-s001.zip › WB-supplementary S3/Figure4-F/HEC_AKT_8bit.tif]

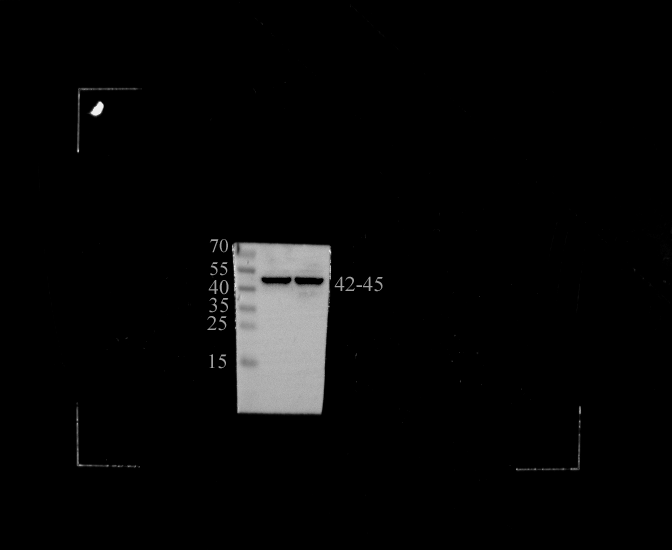

Supplement: Supplementary file 1 [file curroncol-32-00157-s001.zip › WB-supplementary S3/Figure4-F/ISK_1_ACTIN_8bit.png]

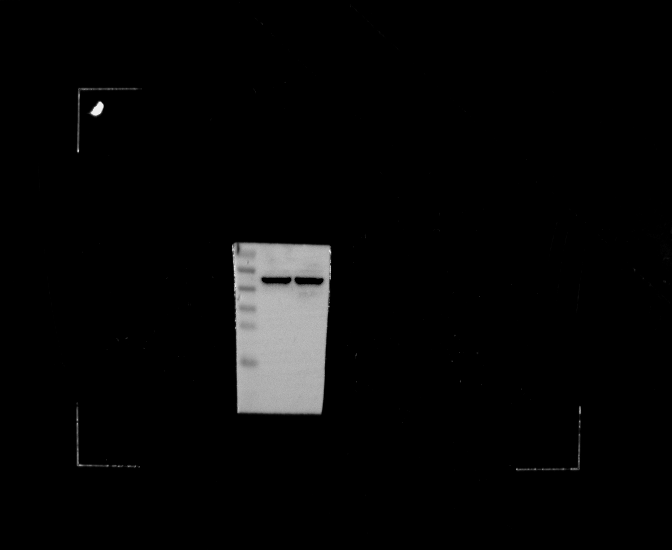

Supplement: Supplementary file 1 [file curroncol-32-00157-s001.zip › WB-supplementary S3/Figure4-F/ISK_1_ACTIN_8bit.tif]

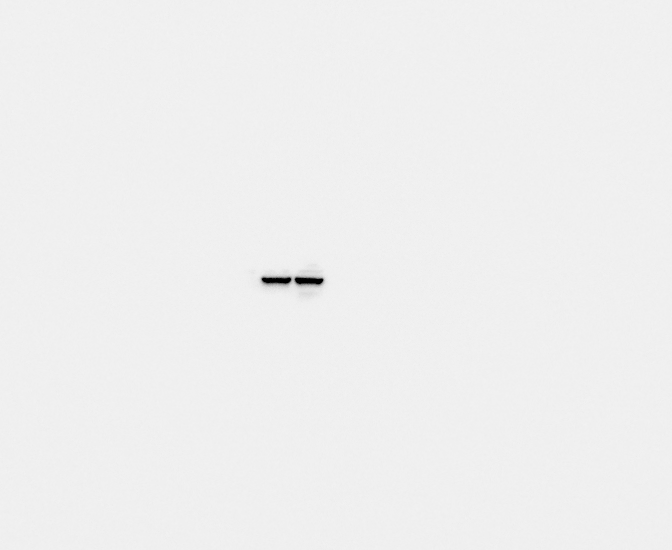

Supplement: Supplementary file 1 [file curroncol-32-00157-s001.zip › WB-supplementary S3/Figure4-F/ISK_1_ACTIN_8bit_8bit.tif]

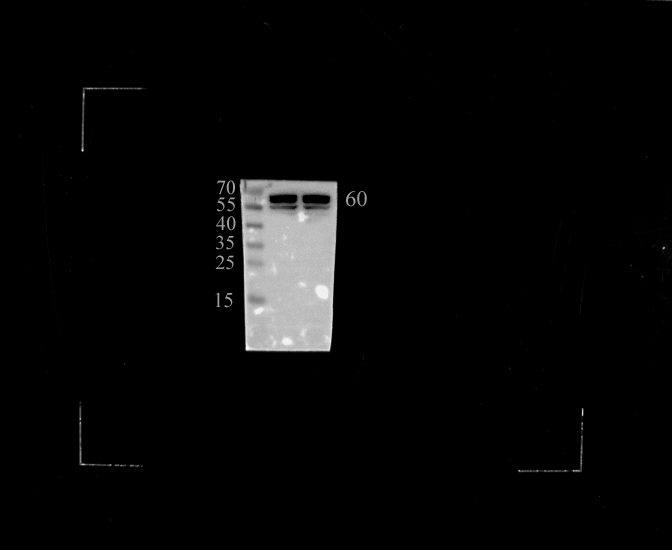

Supplement: Supplementary file 1 [file curroncol-32-00157-s001.zip › WB-supplementary S3/Figure4-F/isk_AKT_8bit.png]

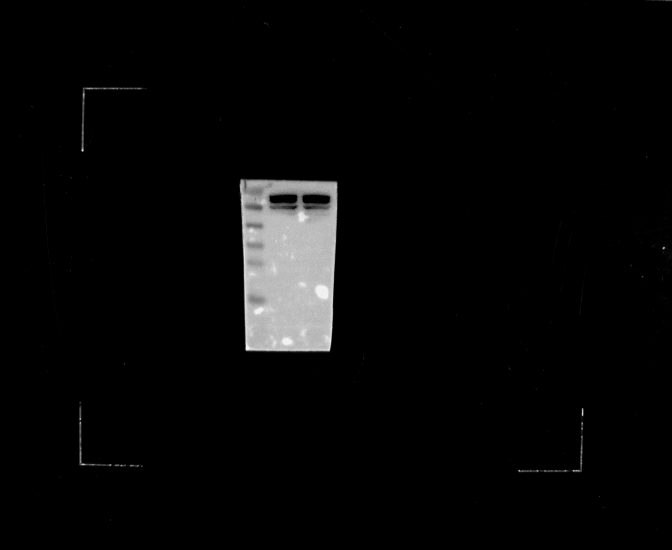

Supplement: Supplementary file 1 [file curroncol-32-00157-s001.zip › WB-supplementary S3/Figure4-F/isk_AKT_8bit.tif]

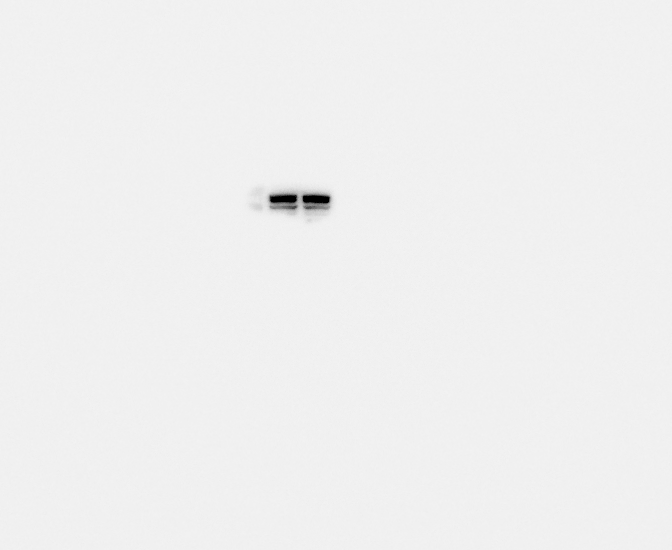

Supplement: Supplementary file 1 [file curroncol-32-00157-s001.zip › WB-supplementary S3/Figure4-F/isk_AKT_8bit_8bit.tif]

55  
40  
35  
25  
15

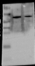

54

Supplement: Supplementary file 1 [file curroncol-32-00157-s001.zip › WB-supplementary S3/Figure4-H/HEC_P_PI3K _8bit.pdf]

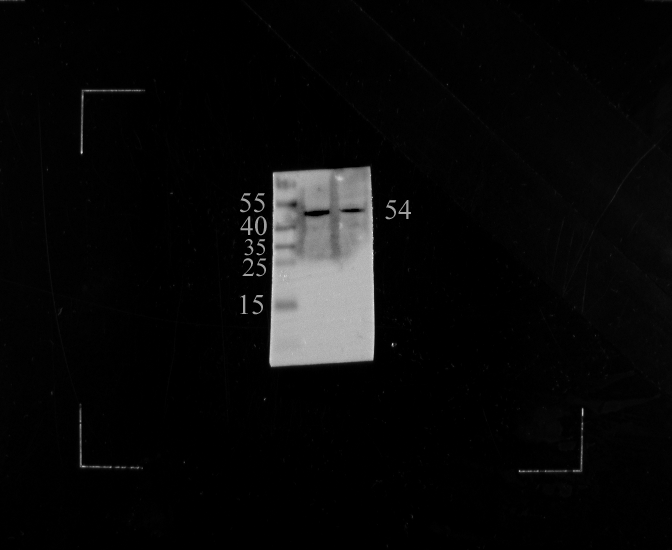

Supplement: Supplementary file 1 [file curroncol-32-00157-s001.zip › WB-supplementary S3/Figure4-H/HEC_P_PI3K _8bit.png]

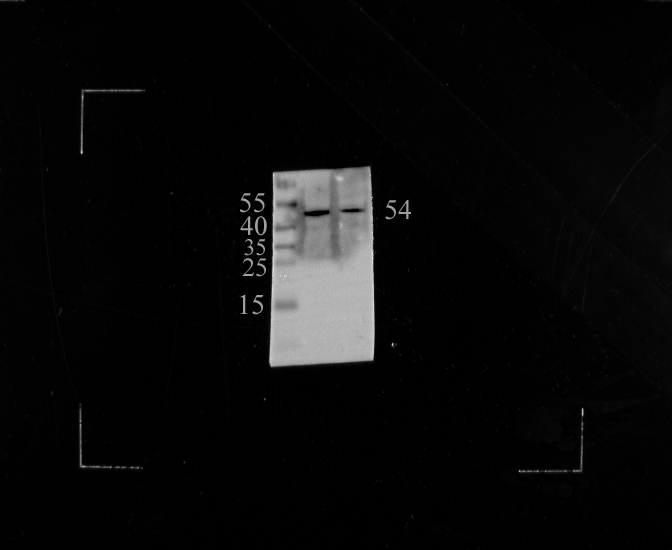

Supplement: Supplementary file 1 [file curroncol-32-00157-s001.zip › WB-supplementary S3/Figure4-H/HEC_P_PI3K _8bit.tif]

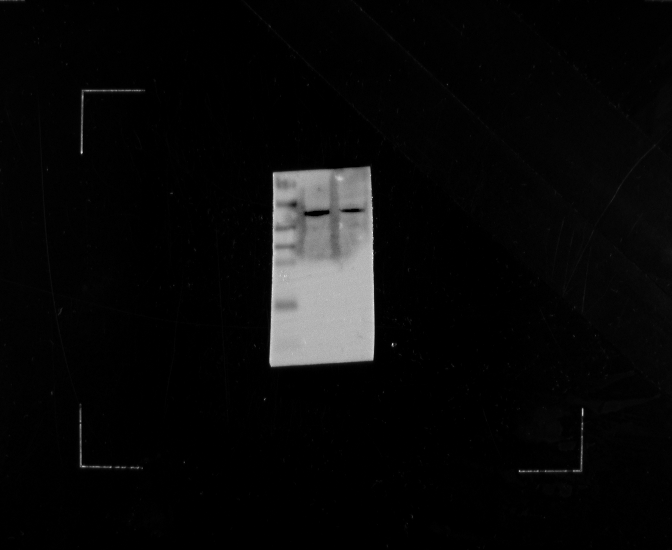

Supplement: Supplementary file 1 [file curroncol-32-00157-s001.zip › WB-supplementary S3/Figure4-H/HEC_P_PI3K_8bit.tif]

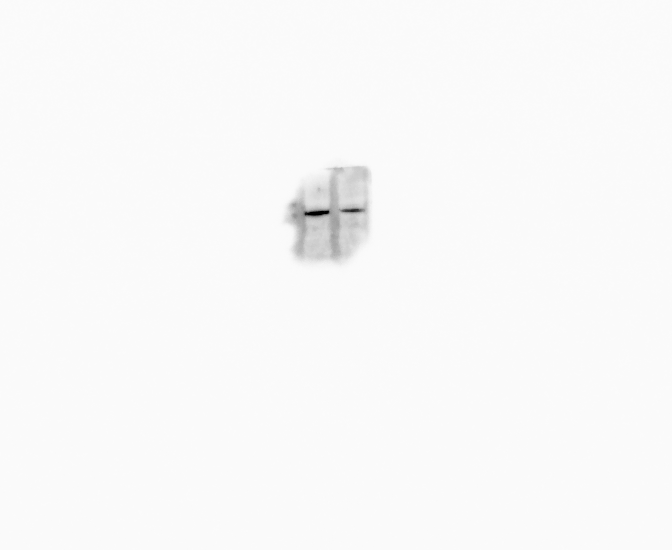

Supplement: Supplementary file 1 [file curroncol-32-00157-s001.zip › WB-supplementary S3/Figure4-H/HEC_P_PI3K_8bit_8bit.tif]

70  
55  
40  
35  
25

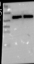

42-45

Supplement: Supplementary file 1 [file curroncol-32-00157-s001.zip › WB-supplementary S3/Figure4-H/HEC_P_PI3K_ACTIN _20241223_114416_00.00.230.clx_8bit_8bit.pdf]

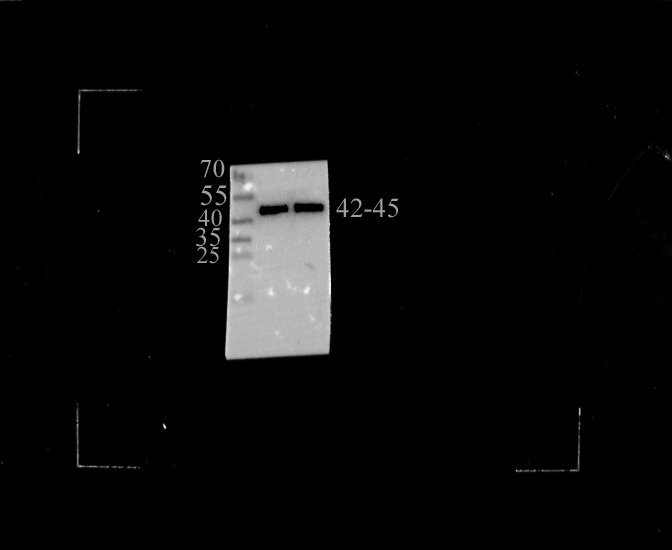

Supplement: Supplementary file 1 [file curroncol-32-00157-s001.zip › WB-supplementary S3/Figure4-H/HEC_P_PI3K_ACTIN _20241223_114416_00.00.230.clx_8bit_8bit.png]

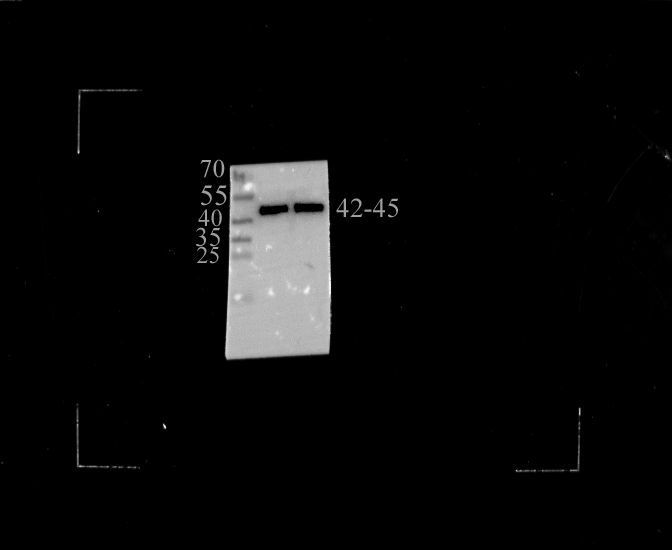

Supplement: Supplementary file 1 [file curroncol-32-00157-s001.zip › WB-supplementary S3/Figure4-H/HEC_P_PI3K_ACTIN _20241223_114416_00.00.230.clx_8bit_8bit.tif]

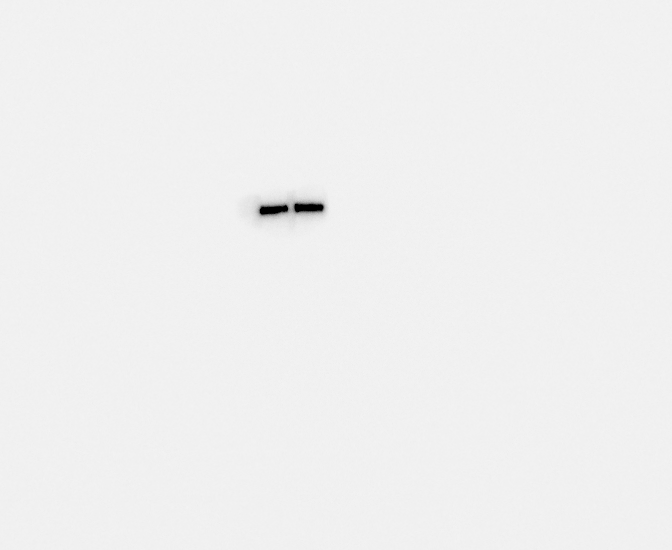

Supplement: Supplementary file 1 [file curroncol-32-00157-s001.zip › WB-supplementary S3/Figure4-H/HEC_P_PI3K_ACTIN_20241223_114416_00.00.230.clx_8bit.tif]

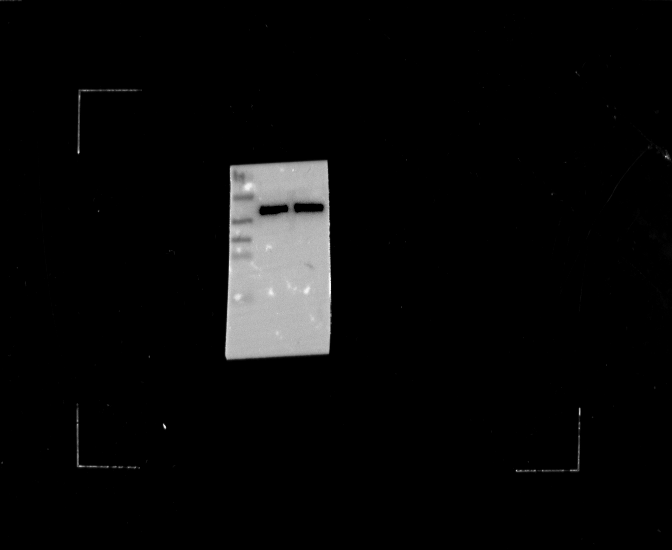

Supplement: Supplementary file 1 [file curroncol-32-00157-s001.zip › WB-supplementary S3/Figure4-H/HEC_P_PI3K_ACTIN_20241223_114416_00.00.230.clx_8bit_8bit.tif]

23405

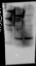

24

Supplement: Supplementary file 1 [file curroncol-32-00157-s001.zip › WB-supplementary S3/Figure4-H/ISK_P_PI3K _8bit.pdf]

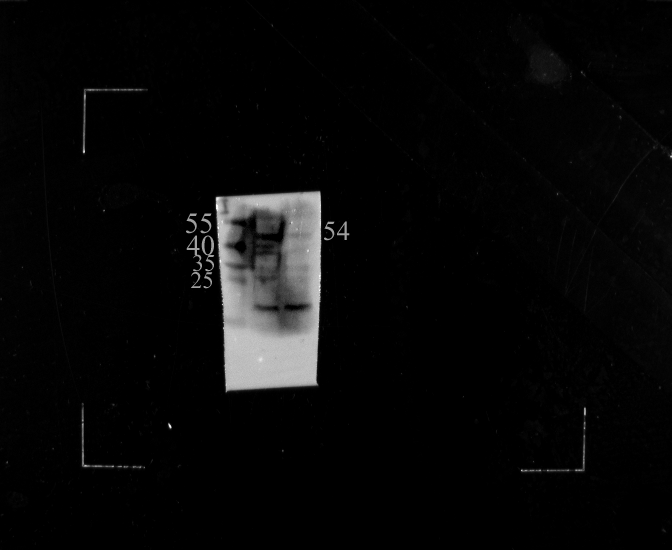

Supplement: Supplementary file 1 [file curroncol-32-00157-s001.zip › WB-supplementary S3/Figure4-H/ISK_P_PI3K _8bit.png]

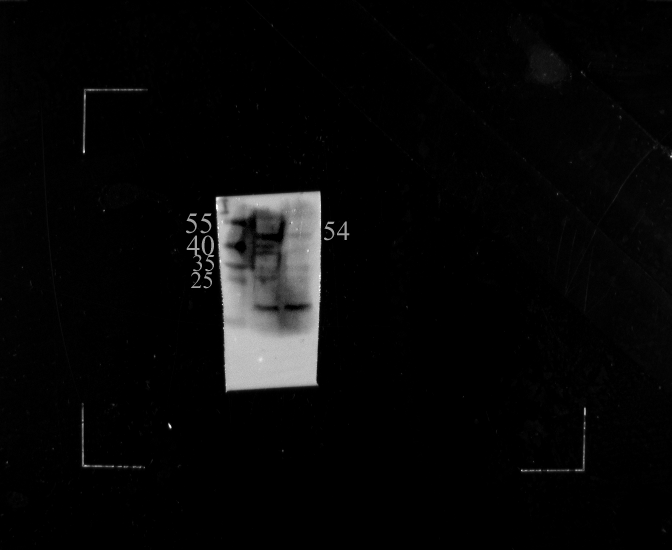

Supplement: Supplementary file 1 [file curroncol-32-00157-s001.zip › WB-supplementary S3/Figure4-H/ISK_P_PI3K _8bit.tif]

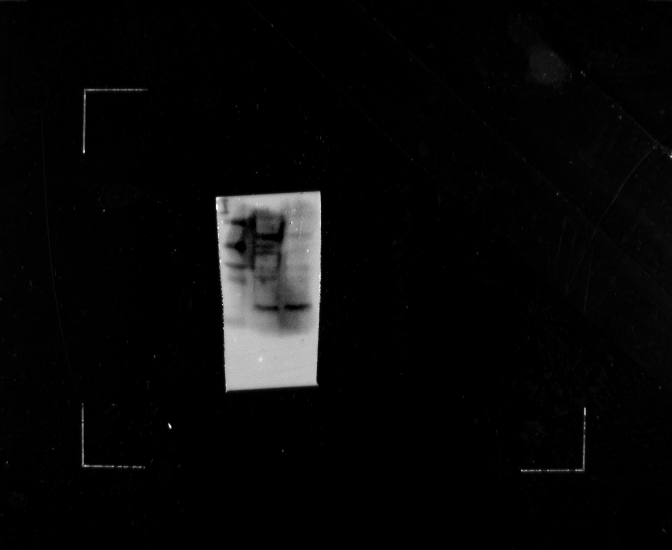

Supplement: Supplementary file 1 [file curroncol-32-00157-s001.zip › WB-supplementary S3/Figure4-H/ISK_P_PI3K_8bit.tif]

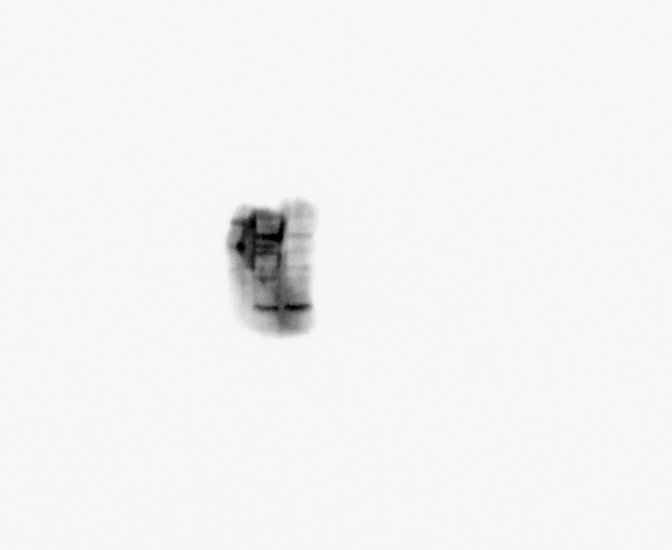

Supplement: Supplementary file 1 [file curroncol-32-00157-s001.zip › WB-supplementary S3/Figure4-H/ISK_P_PI3K_8bit_8bit.tif]

55  
40  
25

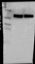

42-45

Supplement: Supplementary file 1 [file curroncol-32-00157-s001.zip › WB-supplementary S3/Figure4-H/ISK_P_PI3K_ACTIN _8bit_8bit.pdf]

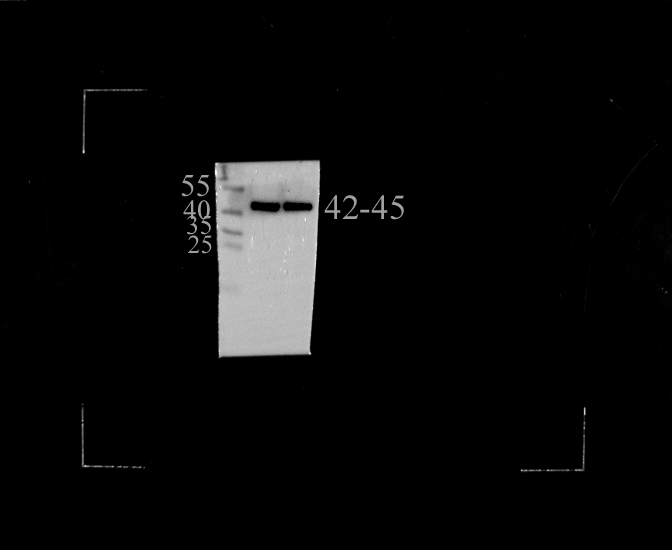

Supplement: Supplementary file 1 [file curroncol-32-00157-s001.zip › WB-supplementary S3/Figure4-H/ISK_P_PI3K_ACTIN _8bit_8bit.png]

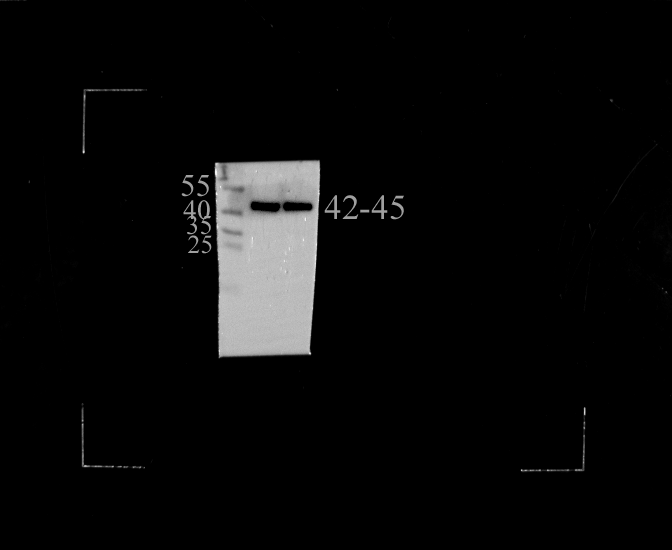

Supplement: Supplementary file 1 [file curroncol-32-00157-s001.zip › WB-supplementary S3/Figure4-H/ISK_P_PI3K_ACTIN _8bit_8bit.tif]

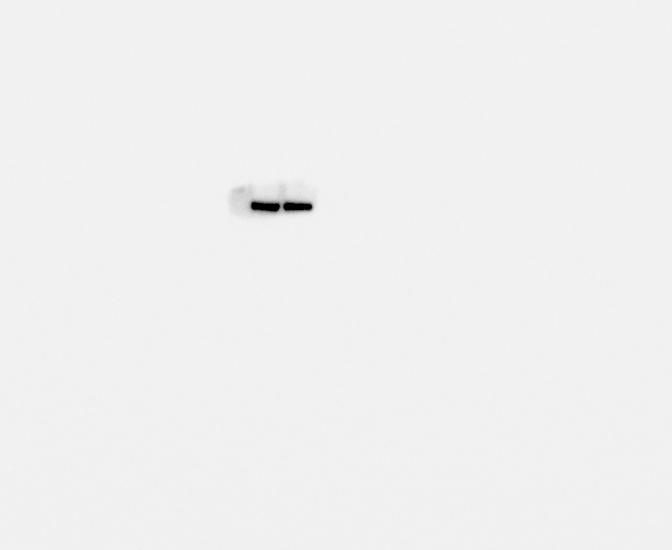

Supplement: Supplementary file 1 [file curroncol-32-00157-s001.zip › WB-supplementary S3/Figure4-H/ISK_P_PI3K_ACTIN_8bit.tif]

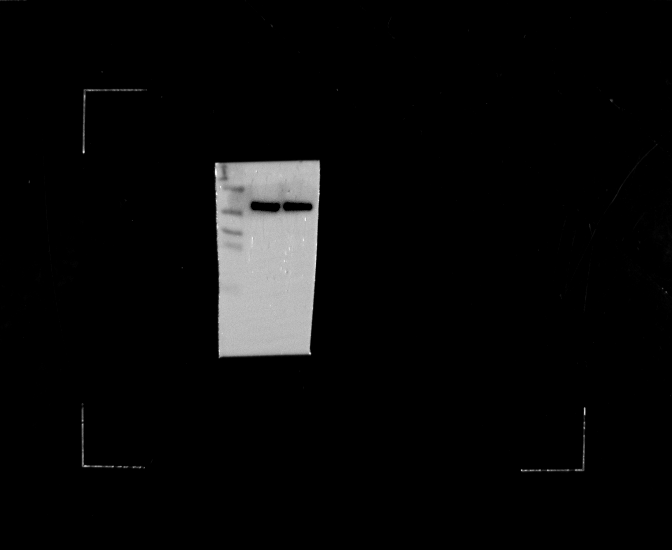

Supplement: Supplementary file 1 [file curroncol-32-00157-s001.zip › WB-supplementary S3/Figure4-H/ISK_P_PI3K_ACTIN_8bit_8bit.tif]

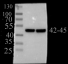

Supplement: Supplementary file 1 [file curroncol-32-00157-s001.zip › WB-supplementary S3/Figure4-J/HEC-pi3kACTIN7——_8bit_8bit.pdf]

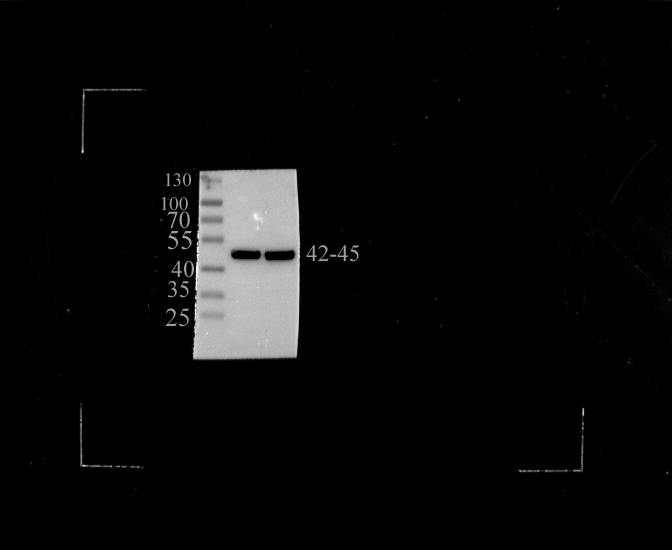

Supplement: Supplementary file 1 [file curroncol-32-00157-s001.zip › WB-supplementary S3/Figure4-J/HEC-pi3kACTIN7——_8bit_8bit.png]

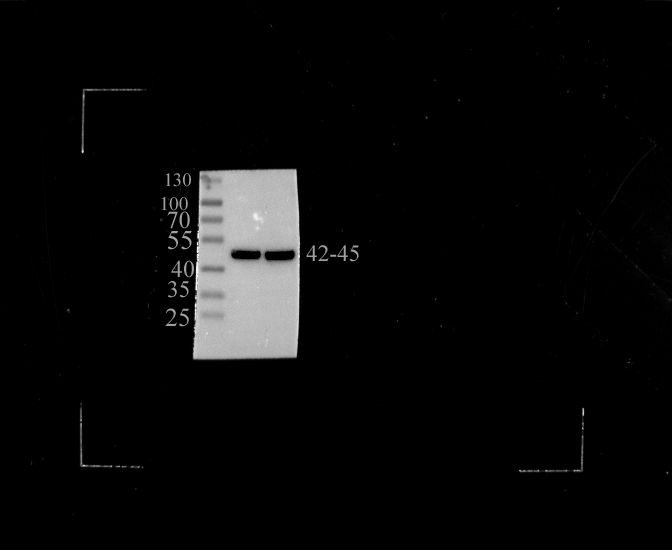

Supplement: Supplementary file 1 [file curroncol-32-00157-s001.zip › WB-supplementary S3/Figure4-J/HEC-pi3kACTIN7——_8bit_8bit.tif]

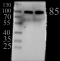

Supplement: Supplementary file 1 [file curroncol-32-00157-s001.zip › WB-supplementary S3/Figure4-J/HEC_PI3K720241228_132410_06.17.358_8bit.pdf]

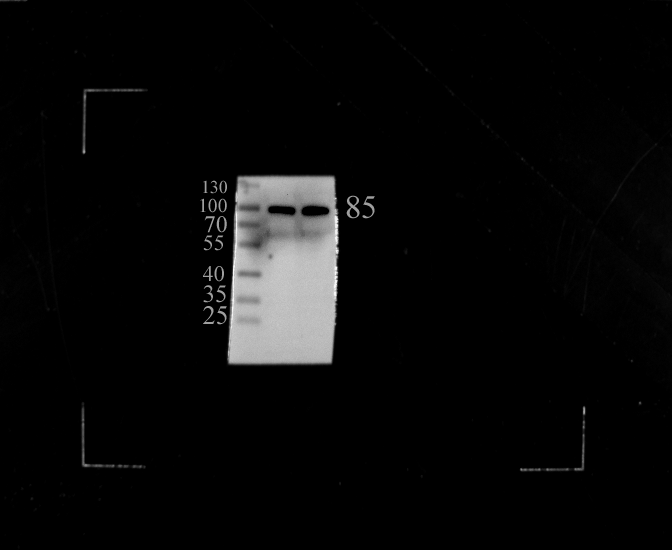

Supplement: Supplementary file 1 [file curroncol-32-00157-s001.zip › WB-supplementary S3/Figure4-J/HEC_PI3K720241228_132410_06.17.358_8bit.png]

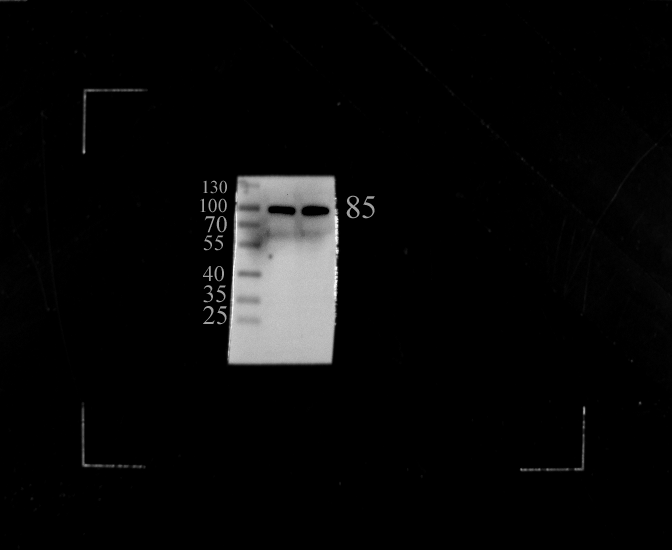

Supplement: Supplementary file 1 [file curroncol-32-00157-s001.zip › WB-supplementary S3/Figure4-J/HEC_PI3K720241228_132410_06.17.358_8bit.tif]

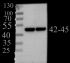

Supplement: Supplementary file 1 [file curroncol-32-00157-s001.zip › WB-supplementary S3/Figure4-J/ISK-pi3kACTIN5——_8bit_8bit.pdf]

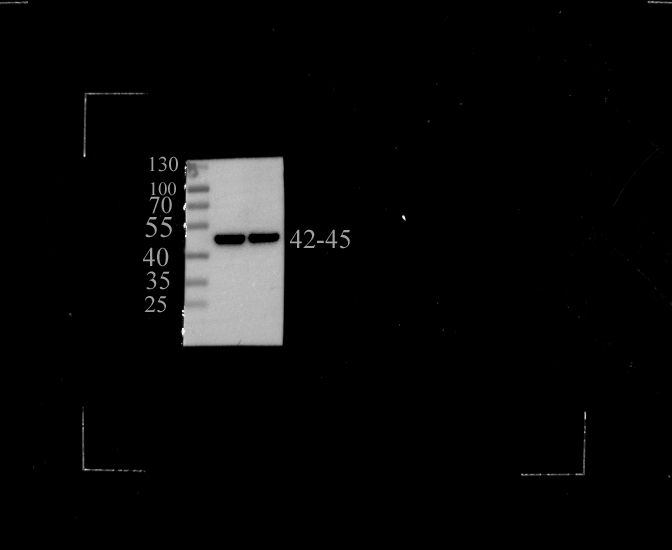

Supplement: Supplementary file 1 [file curroncol-32-00157-s001.zip › WB-supplementary S3/Figure4-J/ISK-pi3kACTIN5——_8bit_8bit.png]

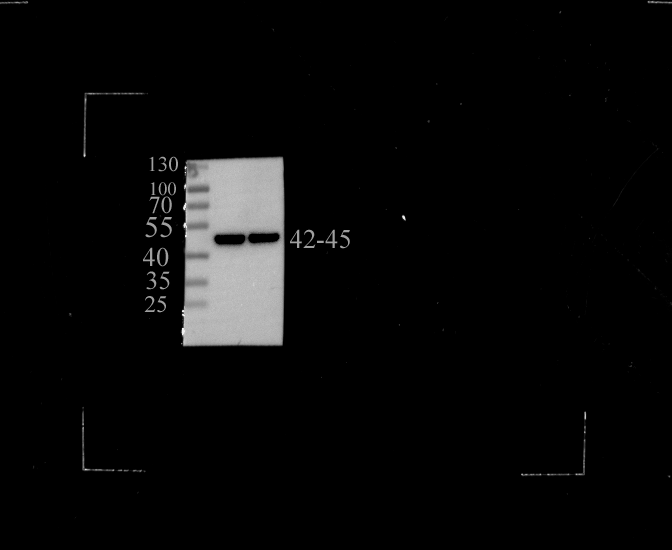

Supplement: Supplementary file 1 [file curroncol-32-00157-s001.zip › WB-supplementary S3/Figure4-J/ISK-pi3kACTIN5——_8bit_8bit.tif]

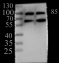

Supplement: Supplementary file 1 [file curroncol-32-00157-s001.zip › WB-supplementary S3/Figure4-J/ISK_PI3K5_8bit.pdf]

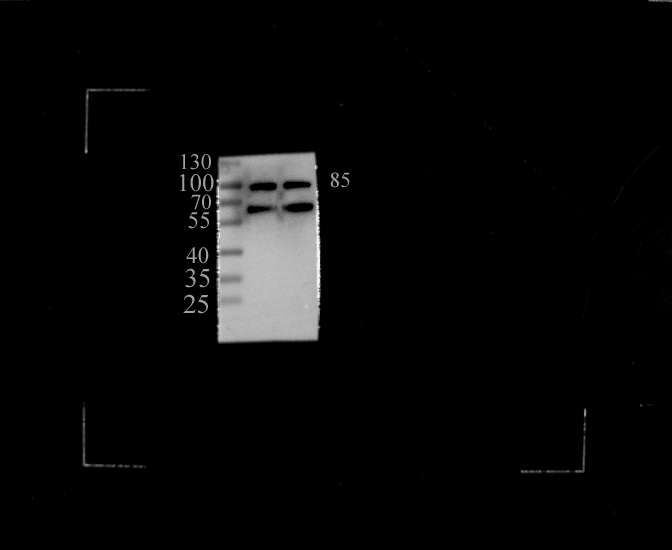

Supplement: Supplementary file 1 [file curroncol-32-00157-s001.zip › WB-supplementary S3/Figure4-J/ISK_PI3K5_8bit.png]

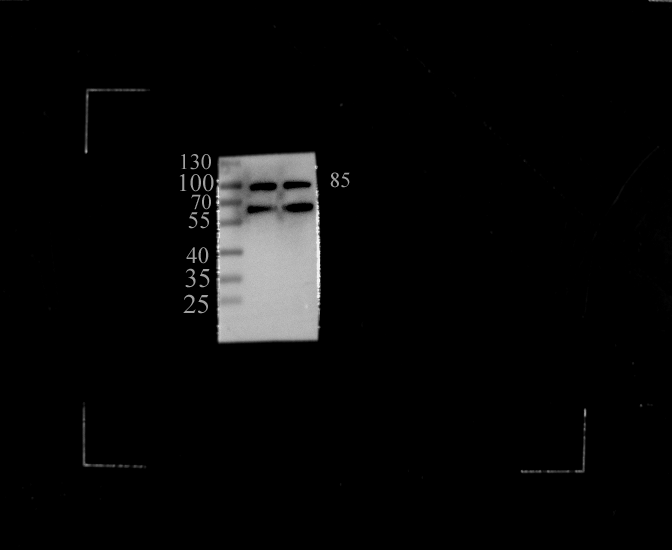

Supplement: Supplementary file 1 [file curroncol-32-00157-s001.zip › WB-supplementary S3/Figure4-J/ISK_PI3K5_8bit.tif]

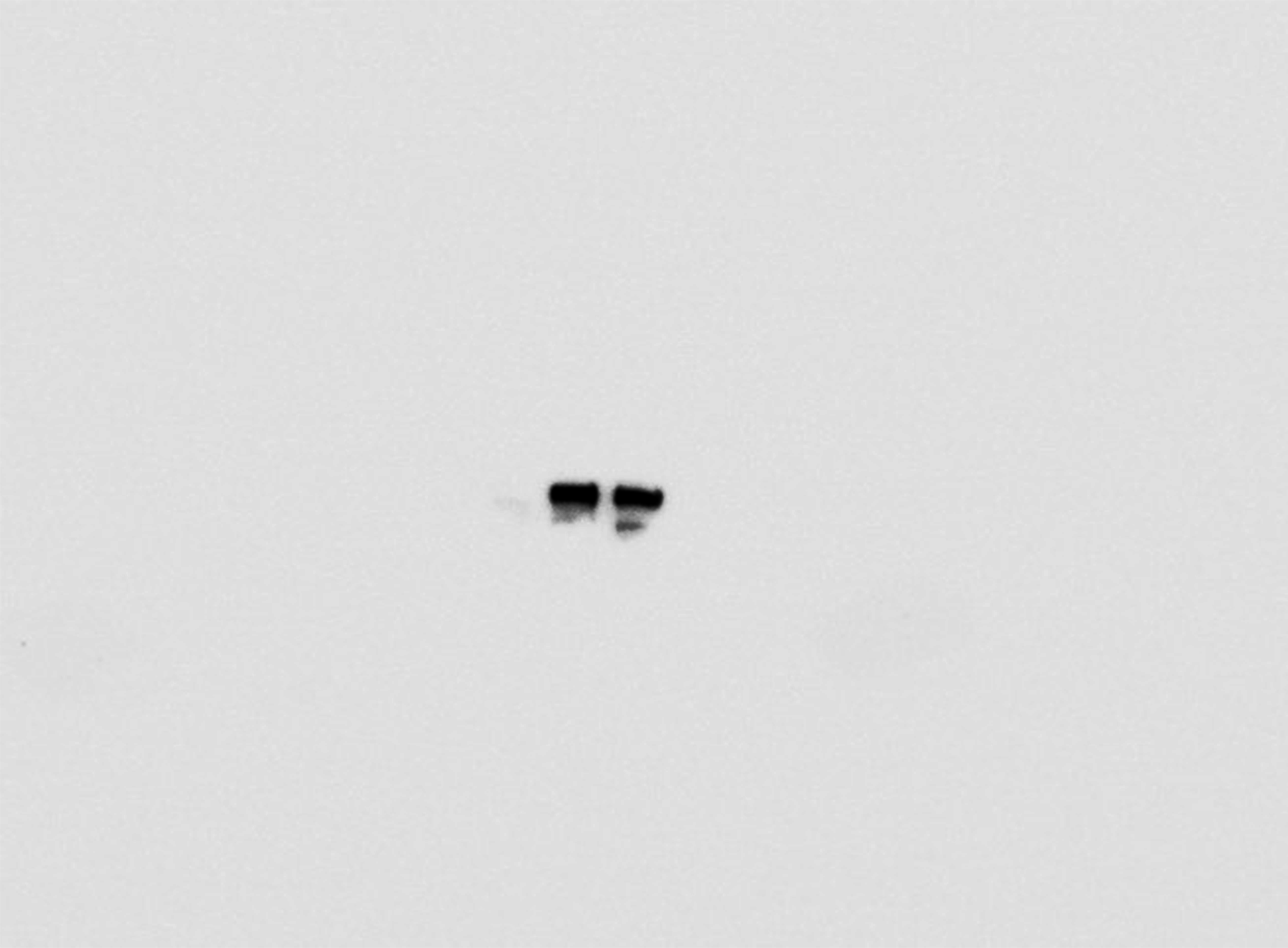

Supplement: Supplementary file 1 [file curroncol-32-00157-s001.zip › WB-supplementary S3/Figure4-L/HEC-m-Tor.png]

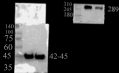

Supplement: Supplementary file 1 [file curroncol-32-00157-s001.zip › WB-supplementary S3/Figure4-L/HEC_MTOR_ACTIN _8bit_8bit_8bit 2.pdf]

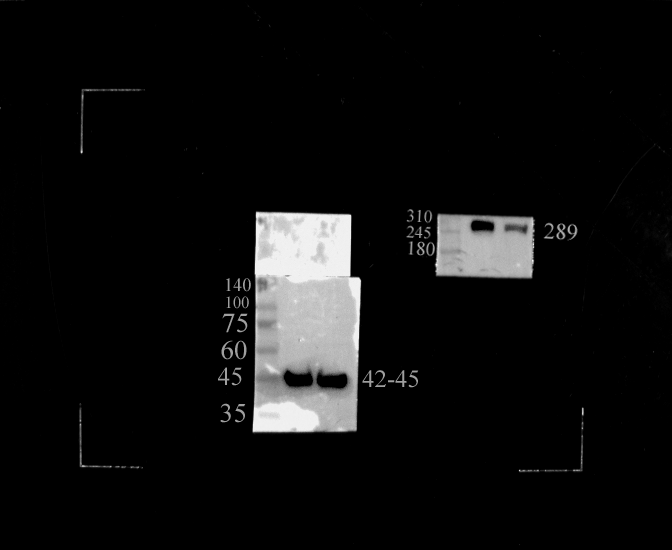

Supplement: Supplementary file 1 [file curroncol-32-00157-s001.zip › WB-supplementary S3/Figure4-L/HEC_MTOR_ACTIN _8bit_8bit_8bit 2.png]

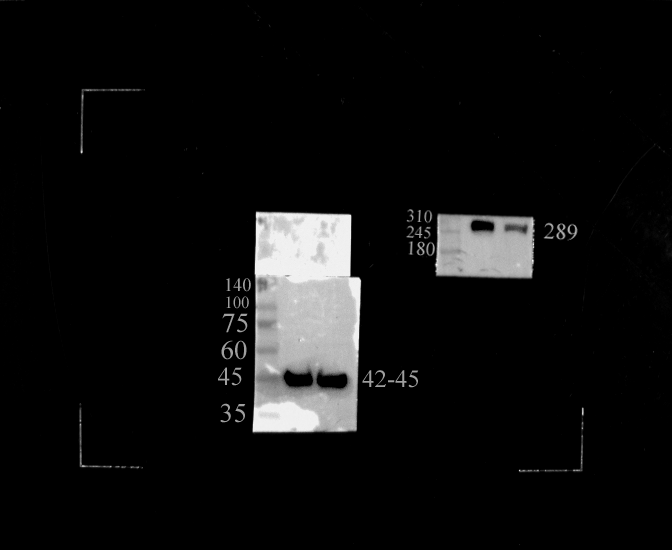

Supplement: Supplementary file 1 [file curroncol-32-00157-s001.zip › WB-supplementary S3/Figure4-L/HEC_MTOR_ACTIN _8bit_8bit_8bit 2.tif]

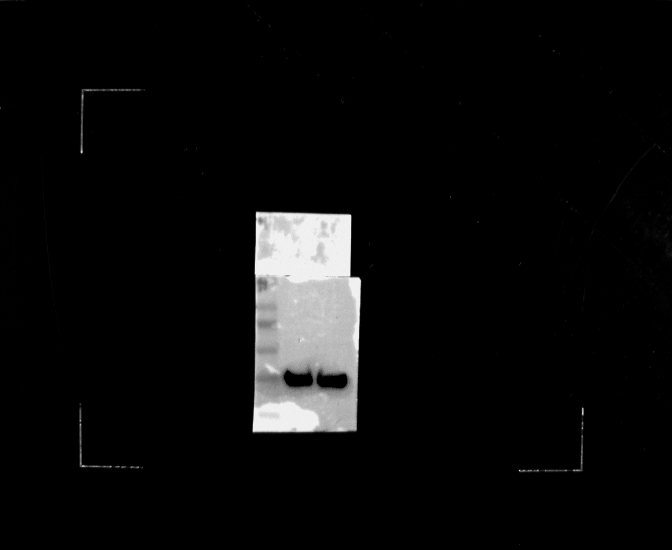

Supplement: Supplementary file 1 [file curroncol-32-00157-s001.zip › WB-supplementary S3/Figure4-L/HEC_MTOR_ACTIN_8bit_8bit_8bit 2.tif]

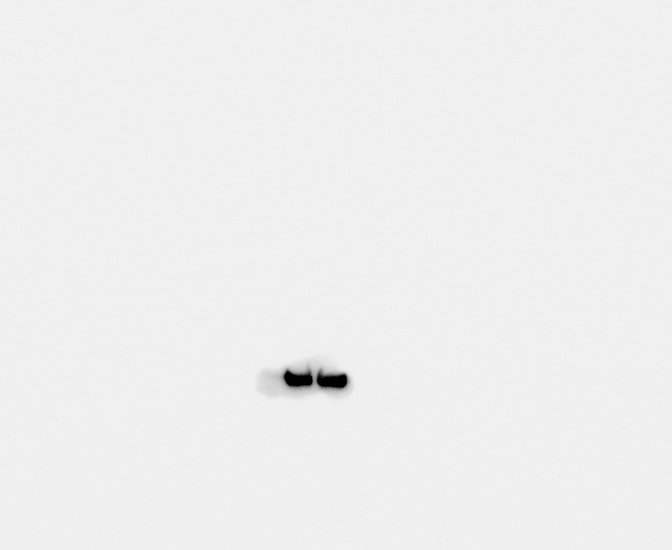

Supplement: Supplementary file 1 [file curroncol-32-00157-s001.zip › WB-supplementary S3/Figure4-L/HEC_MTOR_ACTIN_8bit_8bit_8bit_8bit.tif]

318  
243  
180

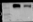

239

Supplement: Supplementary file 1 [file curroncol-32-00157-s001.zip › WB-supplementary S3/Figure4-L/HEC_P_MTOR _8bit_8bit_8bit_8bit_8bit_8bit_8bit.pdf]

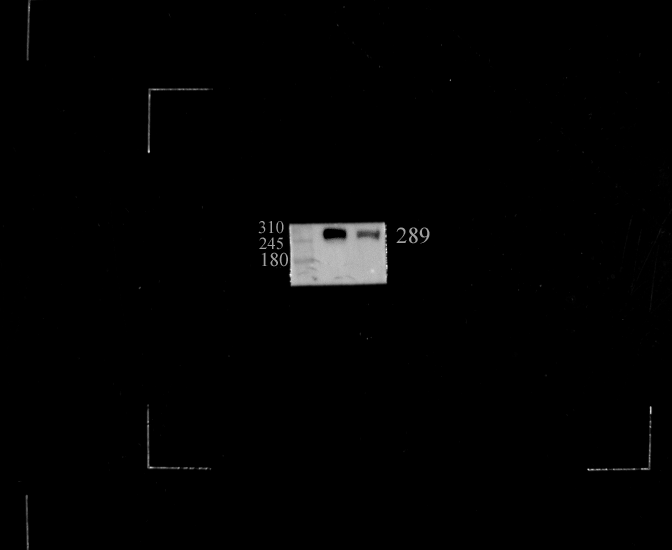

Supplement: Supplementary file 1 [file curroncol-32-00157-s001.zip › WB-supplementary S3/Figure4-L/HEC_P_MTOR _8bit_8bit_8bit_8bit_8bit_8bit_8bit.png]

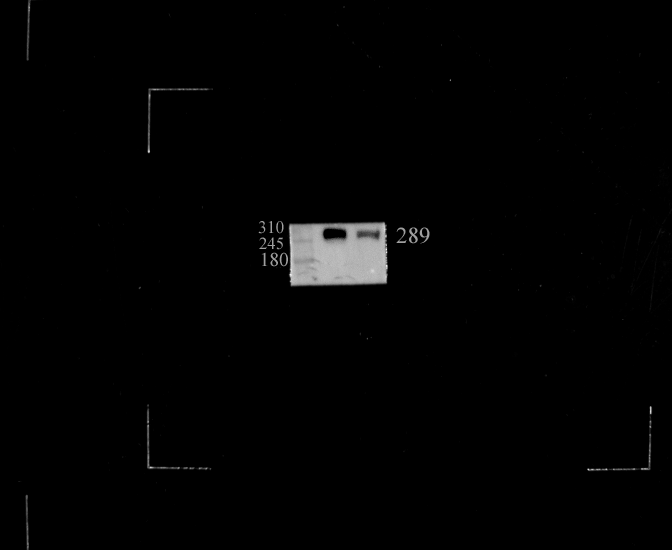

Supplement: Supplementary file 1 [file curroncol-32-00157-s001.zip › WB-supplementary S3/Figure4-L/HEC_P_MTOR _8bit_8bit_8bit_8bit_8bit_8bit_8bit.tif]

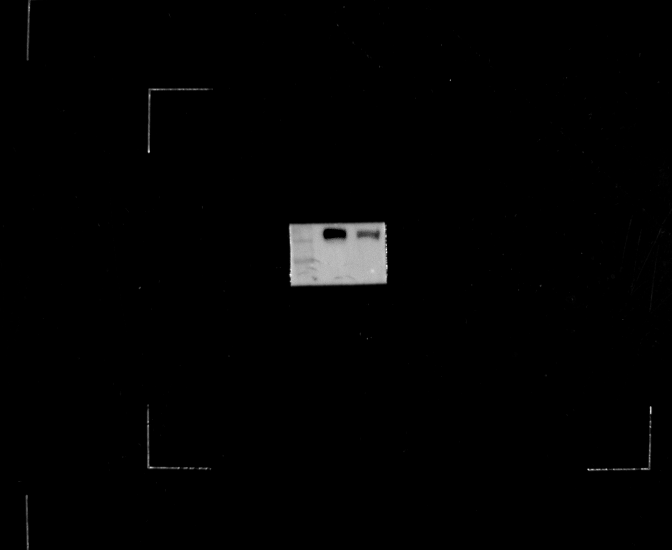

Supplement: Supplementary file 1 [file curroncol-32-00157-s001.zip › WB-supplementary S3/Figure4-L/HEC_P_MTOR_8bit_8bit_8bit_8bit_8bit_8bit_8bit.tif]

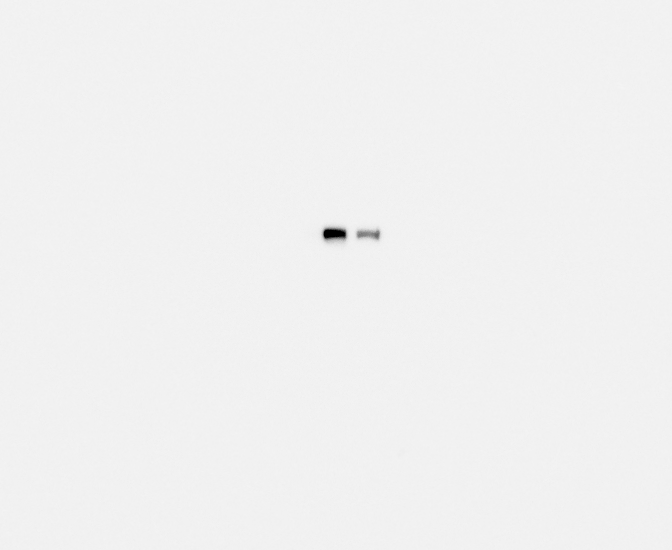

Supplement: Supplementary file 1 [file curroncol-32-00157-s001.zip › WB-supplementary S3/Figure4-L/HEC_P_MTOR_8bit_8bit_8bit_8bit_8bit_8bit_8bit_8bit.tif]

310  
245  
180

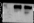

289

310  
245  
180

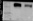

289

Supplement: Supplementary file 1 [file curroncol-32-00157-s001.zip › WB-supplementary S3/Figure4-L/HE_MTOR _8bit_8bit_8bit_8bit.pdf]

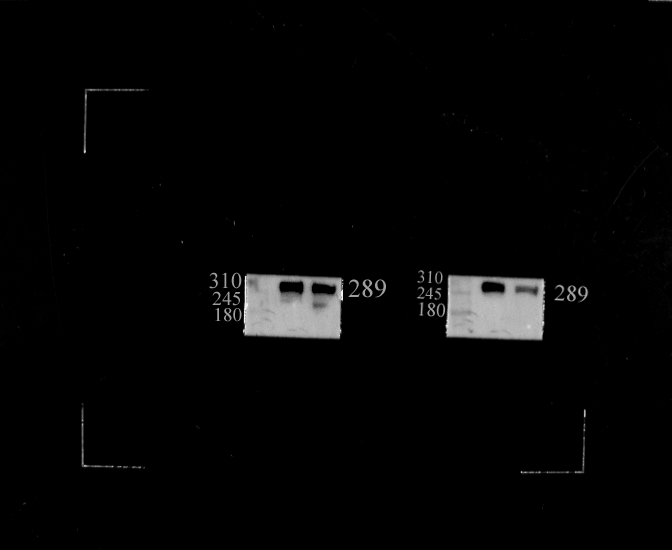

Supplement: Supplementary file 1 [file curroncol-32-00157-s001.zip › WB-supplementary S3/Figure4-L/HE_MTOR _8bit_8bit_8bit_8bit.png]

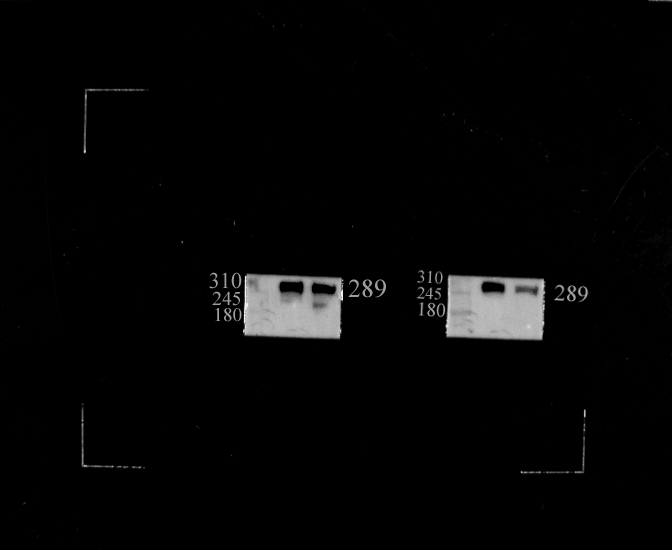

Supplement: Supplementary file 1 [file curroncol-32-00157-s001.zip › WB-supplementary S3/Figure4-L/HE_MTOR _8bit_8bit_8bit_8bit.tif]

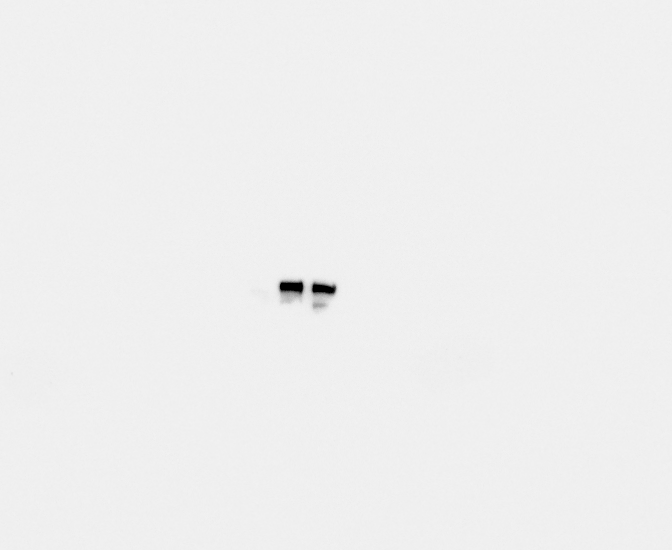

Supplement: Supplementary file 1 [file curroncol-32-00157-s001.zip › WB-supplementary S3/Figure4-L/HE_MTOR_8bit_8bit_8bit.tif]

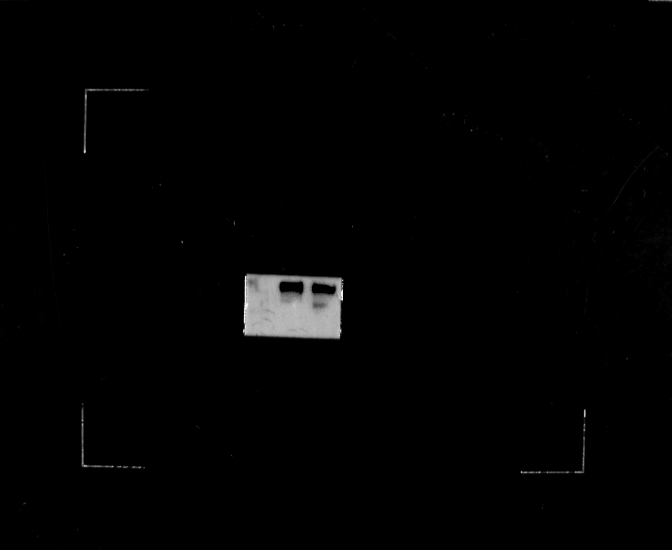

Supplement: Supplementary file 1 [file curroncol-32-00157-s001.zip › WB-supplementary S3/Figure4-L/HE_MTOR_8bit_8bit_8bit_8bit.tif]

310  
245  
180

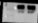

289

310  
245  
180

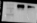

289

Supplement: Supplementary file 1 [file curroncol-32-00157-s001.zip › WB-supplementary S3/Figure4-L/ISK_MTOR _8bit_8bit.pdf]

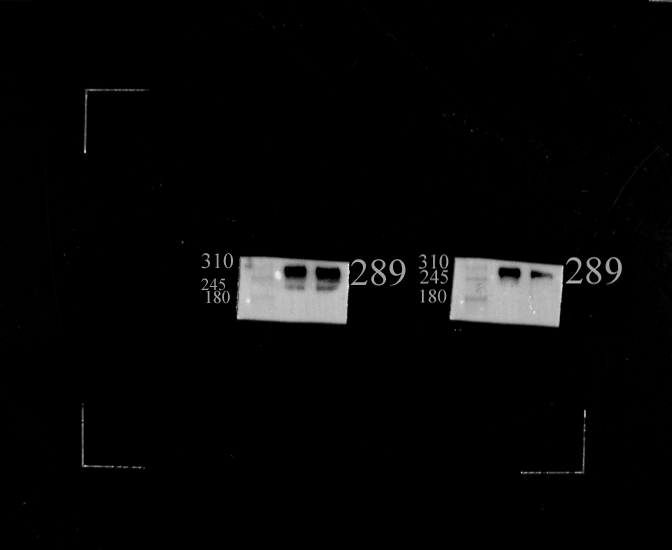

Supplement: Supplementary file 1 [file curroncol-32-00157-s001.zip › WB-supplementary S3/Figure4-L/ISK_MTOR _8bit_8bit.png]

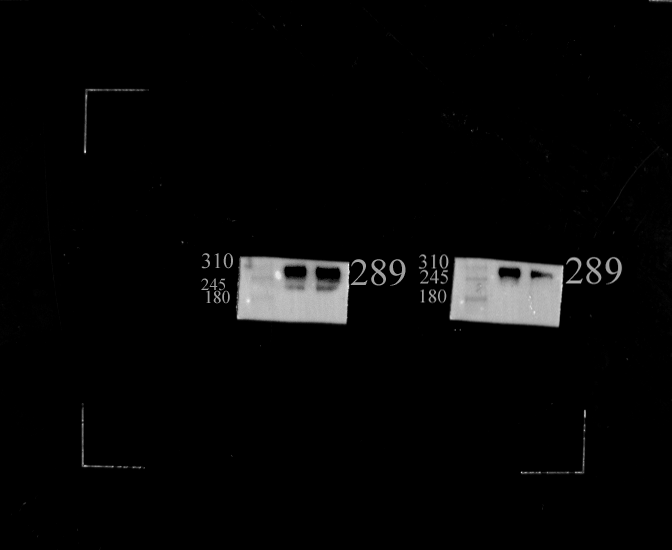

Supplement: Supplementary file 1 [file curroncol-32-00157-s001.zip › WB-supplementary S3/Figure4-L/ISK_MTOR _8bit_8bit.tif]

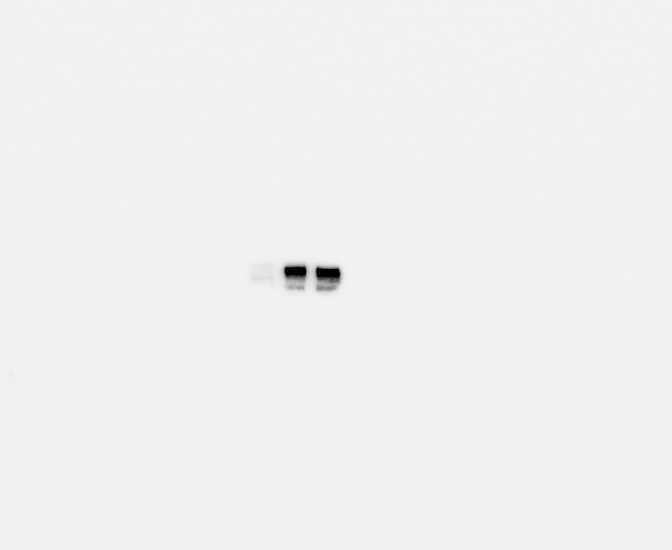

Supplement: Supplementary file 1 [file curroncol-32-00157-s001.zip › WB-supplementary S3/Figure4-L/ISK_MTOR_8bit.tif]

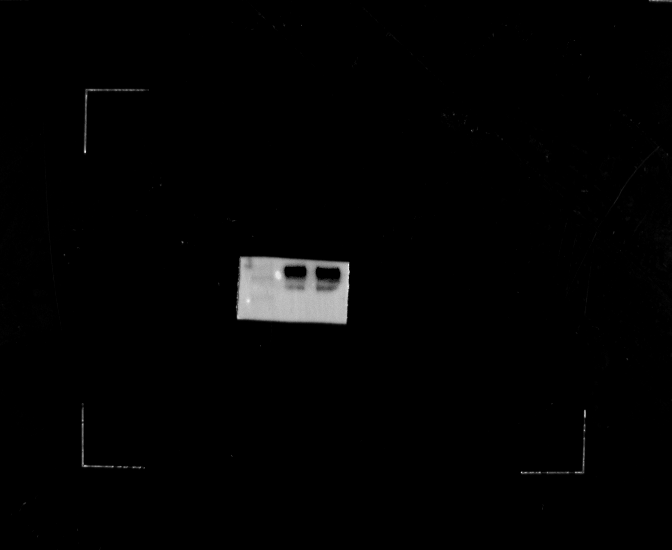

Supplement: Supplementary file 1 [file curroncol-32-00157-s001.zip › WB-supplementary S3/Figure4-L/ISK_MTOR_8bit_8bit.tif]

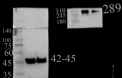

Supplement: Supplementary file 1 [file curroncol-32-00157-s001.zip › WB-supplementary S3/Figure4-L/ISK_MTOR_ACTIN _8bit_8bit_8bit_8bit.pdf]

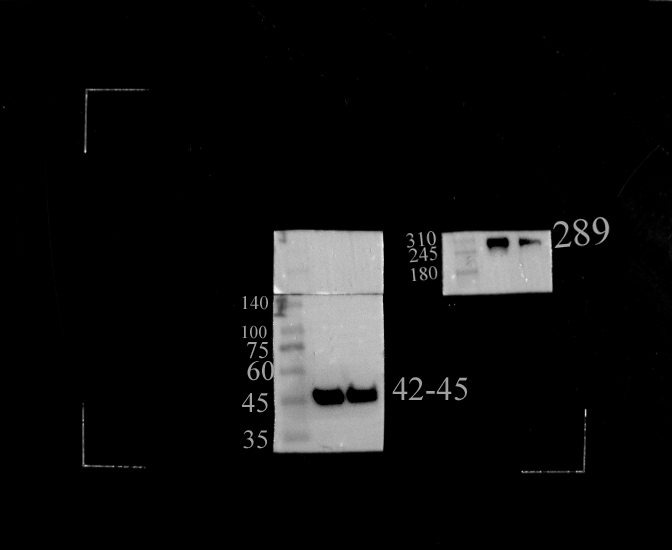

Supplement: Supplementary file 1 [file curroncol-32-00157-s001.zip › WB-supplementary S3/Figure4-L/ISK_MTOR_ACTIN _8bit_8bit_8bit_8bit.png]

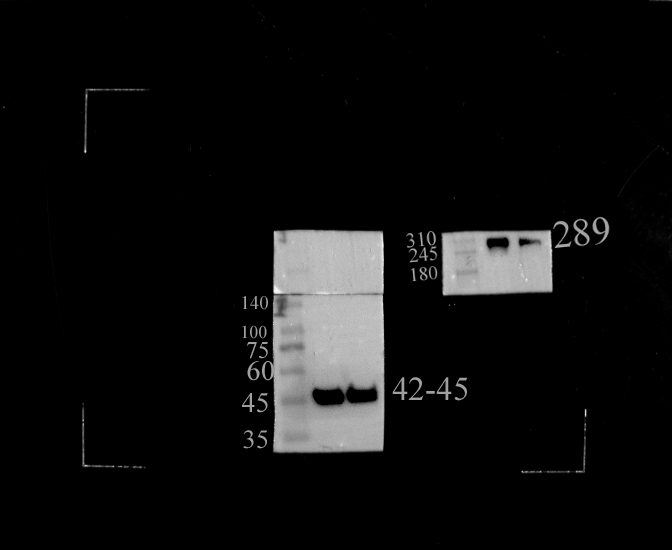

Supplement: Supplementary file 1 [file curroncol-32-00157-s001.zip › WB-supplementary S3/Figure4-L/ISK_MTOR_ACTIN _8bit_8bit_8bit_8bit.tif]

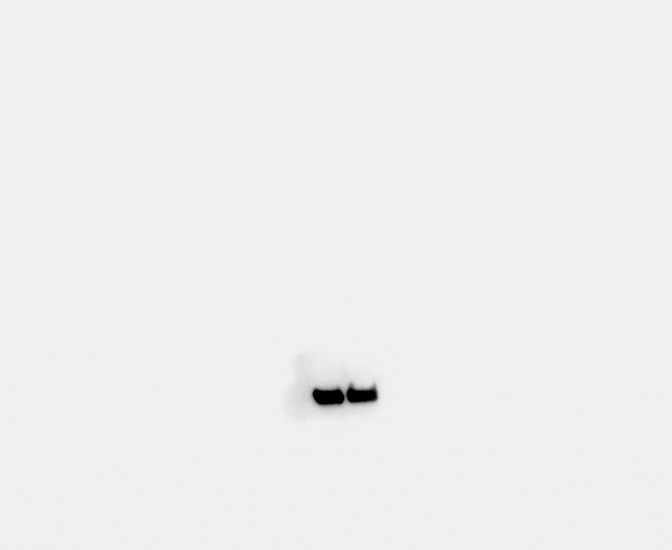

Supplement: Supplementary file 1 [file curroncol-32-00157-s001.zip › WB-supplementary S3/Figure4-L/ISK_MTOR_ACTIN_8bit_8bit_8bit.tif]

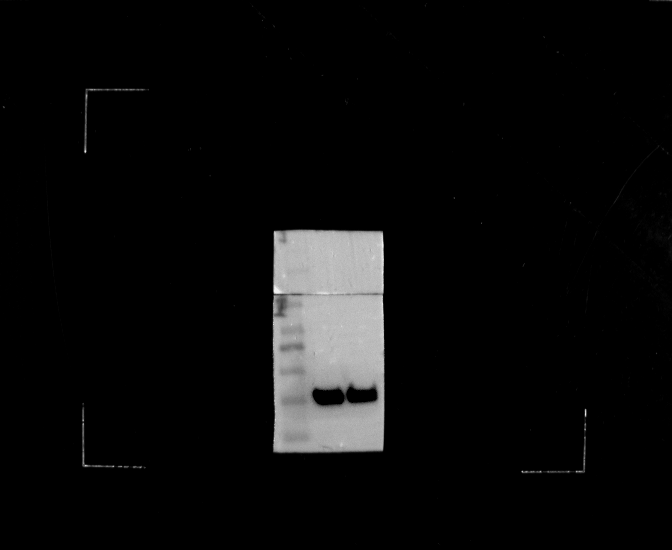

Supplement: Supplementary file 1 [file curroncol-32-00157-s001.zip › WB-supplementary S3/Figure4-L/ISK_MTOR_ACTIN_8bit_8bit_8bit_8bit.tif]

316  
243  
180

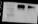

289

Supplement: Supplementary file 1 [file curroncol-32-00157-s001.zip › WB-supplementary S3/Figure4-L/ISK_P_MTOR _8bit_8bit_8bit_8bit.pdf]

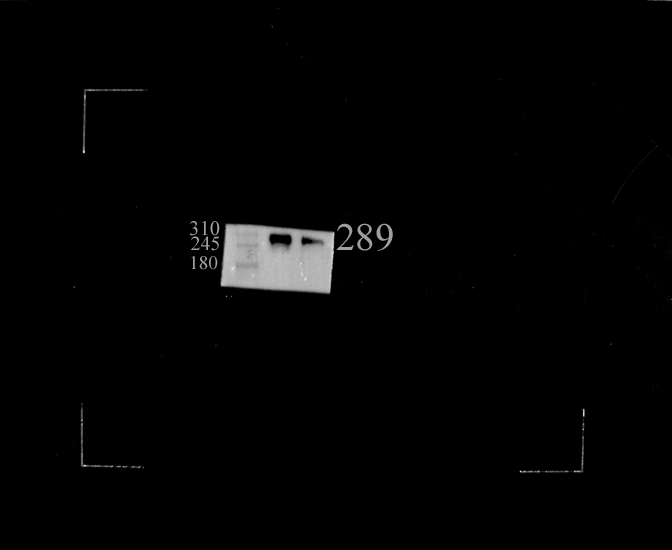

Supplement: Supplementary file 1 [file curroncol-32-00157-s001.zip › WB-supplementary S3/Figure4-L/ISK_P_MTOR _8bit_8bit_8bit_8bit.png]
